# Correlation of structure, function and protein dynamics in GH7 cellobiohydrolases from *Trichoderma atroviride*, *T. reesei* and *T. harzianum*

**DOI:** 10.1186/s13068-017-1006-7

**Published:** 2018-01-13

**Authors:** Anna S. Borisova, Elena V. Eneyskaya, Suvamay Jana, Silke F. Badino, Jeppe Kari, Antonella Amore, Magnus Karlsson, Henrik Hansson, Mats Sandgren, Michael E. Himmel, Peter Westh, Christina M. Payne, Anna A. Kulminskaya, Jerry Ståhlberg

**Affiliations:** 1 Department of Molecular Sciences, Swedish University of Agricultural Sciences, P.O. Box 7015, 750 07 Uppsala, Sweden; 2 B.P. Konstantinov Petersburg Nuclear Physics Institute, National Research Centre “Kurchatov Institute”, Orlova Roscha, Gatchina, Leningrad Region 188300 Russia; 3 Department of Chemical and Materials Engineering, University of Kentucky, 177 F. Paul Anderson Tower, Lexington, KY 40506-0046 USA; 4 Department of Science and Environment, Roskilde University, 1 Universitetsvej, 4000 Roskilde, Denmark; 5 National Renewable Energy Laboratory, Biosciences Center, 15013 Denver West Parkway, Golden, CO 80401 USA; 6 Department of Forest Mycology and Plant Pathology, Swedish University of Agricultural Sciences, P.O. Box 7026, 750 07 Uppsala, Sweden; 7 Department of Medical Physics, Peter the Great St. Petersburg Polytechnic University, Saint Petersburg, Russia; 8 Present Address: Division of Chemical, Bioengineering, Environmental, and Transport Systems, National Science Foundation, Alexandria, VA USA

**Keywords:** Cellobiohydrolase, *Trichoderma atroviride*, *Trichoderma harzianum*, *Trichoderma reesei*, Cellulase engineering

## Abstract

**Background:**

The ascomycete fungus *Trichoderma reesei* is the predominant source of enzymes for industrial conversion of lignocellulose. Its glycoside hydrolase family 7 cellobiohydrolase (GH7 CBH) *Tre*Cel7A constitutes nearly half of the enzyme cocktail by weight and is the major workhorse in the cellulose hydrolysis process. The orthologs from *Trichoderma atroviride* (*Tat*Cel7A) and *Trichoderma harzianum* (*Tha*Cel7A) show high sequence identity with *Tre*Cel7A, ~ 80%, and represent naturally evolved combinations of cellulose-binding tunnel-enclosing loop motifs, which have been suggested to influence intrinsic cellobiohydrolase properties, such as endo-initiation, processivity, and off-rate.

**Results:**

The *Tat*Cel7A, *Tha*Cel7A, and *Tre*Cel7A enzymes were characterized for comparison of function. The catalytic domain of *Tat*Cel7A was crystallized, and two structures were determined: without ligand and with thio-cellotriose in the active site. Initial hydrolysis of bacterial cellulose was faster with *Tat*Cel7A than either *Tha*Cel7A or *Tre*Cel7A. In synergistic saccharification of pretreated corn stover, both *Tat*Cel7A and *Tha*Cel7A were more efficient than *Tre*Cel7A, although *Tat*Cel7A was more sensitive to thermal inactivation. Structural analyses and molecular dynamics (MD) simulations were performed to elucidate important structure/function correlations. Moreover, reverse conservation analysis (RCA) of sequence diversity revealed divergent regions of interest located outside the cellulose-binding tunnel of *Trichoderma* spp. GH7 CBHs.

**Conclusions:**

We hypothesize that the combination of loop motifs is the main determinant for the observed differences in Cel7A activity on cellulosic substrates. Fine-tuning of the loop flexibility appears to be an important evolutionary target in *Trichoderma* spp., a conclusion supported by the RCA data. Our results indicate that, for industrial use, it would be beneficial to combine loop motifs from *Tat*Cel7A with the thermostability features of *Tre*Cel7A. Furthermore, one region implicated in thermal unfolding is suggested as a primary target for protein engineering.

**Electronic supplementary material:**

The online version of this article (10.1186/s13068-017-1006-7) contains supplementary material, which is available to authorized users.

## Background

Cellulolytic fungi are responsible for the majority of the degradation of terrestrial plants, which in turn accounts for most of the Earth’s biomass. In many of these fungi, the major secreted enzymes are glycoside hydrolase family 7 (GH7) cellobiohydrolases (CBH) [1]. GH7 CBHs are the workhorses of cellulose degradation and, thus, play a key role in the recycling of the biosphere. Lignocellulosic biomass is also by far the most abundant renewable carbon source available to humanity for transition from fossil-based to sustainable production of fuels and chemicals. As central as these enzymes are to biomass degradation, they have become the cornerstone of modern industrial enzyme formulations for biofuel processes [2]. As such, GH7 CBHs are the target of intense structural, mechanistic, and engineering studies [3–9].

The ascomycete fungus *T. reesei* is the predominant source of enzymes for industrial lignocellulosic ethanol production, largely because of the development of hyper-producing strains capable of secreting over 50 g/L of protein [2, 10]. The major component, GH7 CBH from *T. reesei* (*Tre*Cel7A), constitutes nearly half of the total protein in the secretome [11]. *Tre*Cel7A is the most extensively studied of GH7s and serves as a model enzyme for GH7 CBHs. About one-third of the known GH7 members are bimodular, having a family 1 carbohydrate-binding module (CBM1) linked to the catalytic domain (CD) by a glycosylated, flexible peptide comprised of about 30 amino acids [12–14]. The first crystal structure of a GH7 catalytic domain (CD) was obtained from *Tre*Cel7A in 1994 [15], and the first structure of a fungal CBM1 was determined by NMR in 1989 [16].

Structurally, GH7 proteins share a β-jelly roll fold with two β-sheets packing face-to-face into a curved β-sandwich. Loop regions extend the edges of the β-sandwich to form a 45 Å-long groove along the entire catalytic domain. CBHs within GH7 are readily distinguished because several loops are further elongated, effectively enclosing the active site in a tunnel. This enables the CBHs to act processively along a cellulose chain and cleave off numerous cellobiose units before detachment from the substrate, which is believed to be key to their efficiency on highly crystalline cellulose [7]. Although often referred to as exoglucanases, CBHs are not true exo-enzymes, in the sense that they do not seem to be exclusively restricted to chain initiation by threading of a chain end through the tunnel. Experiments with *Tre*Cel7A and *Phanerochaete chrysosporium* Cel7D (*Pch*Cel7D) reveal substantial ratios of endo-initiation (40–80%; [17]), suggesting that the tunnel-enclosing loops in these enzymes are sufficiently flexible to open occasionally.

Compared to other GH families, the degree of conservation of GH7 CBHs through evolution is remarkably high. In addition to fungi, GH7 encoding genes are found in very distant branches of the eukaryotic tree of life, such as Amoebozoa, Oomycetes, Dinoflagellates and Crustaceans. GH7 encoding genes have not been found in any prokaryote so far [18, 19]. The sequence identity is over 40% between organisms that diverged more than 1 billion years ago, suggesting that GH7 CBHs cannot accommodate a broad sequence space for primary function [18]. Differences are primarily found in loops and surface regions distant from the active site. However, there are also small variations in the length and sequence of loop regions along the cellulose-binding path that will affect the dynamics of loop regions and the accessibility of the active site [3, 20, 21]. These variations may in turn influence key enzymatic properties, such as processivity, product inhibition, endo-initiation and rate of substrate dissociation [8, 17].

*Trichoderma* species have attracted attention as alternative enzyme sources [22–25], among other reasons. For example, whereas *T. reesei* is a weak mycoparasite and is adapted to a saprotrophic lifestyle as a wood degrader [26], most *Trichoderma* spp. are described as mycoparasitic fungi and several have garnered interest as powerful biocontrol agents (BCA) against pathogenic fungi [27]. Such BCA fungi include *T. harzianum* and *T. atroviride*. These fungi have a cosmopolitan distribution and are commonly found in soil in both tropical and temperate climates. Both are considered to have a broad ecological opportunistic lifestyle, where they can live as saprotrophs of dead organic matter (plant, fungal, and animal) but also interact with plants and other fungi as mutualistic symbionts and necrotrophic mycoparasites [28]. They are widely studied for their capacity to produce antibiotics, parasitize other fungi, and control diseases caused by plant pathogenic microorganisms [29]. The cellulolytic secretomes of both *T. harzianum* and *T. atroviride* have higher β-glucosidase activity and are competitive with that of *T. reesei* on lignocellulose [22, 24, 30].

The GH7 CBHs of *T. harzianum* (*Tha*Cel7A) and *T. atroviride* (*Tat*Cel7A) share the GH7_CD-linker-CBM1 bimodular organization and are very similar to each other and to *Tre*Cel7A, with ~ 80% pairwise sequence identities. Whereas the characterization of *Tat*Cel7A has not previously been reported, the *Tha*Cel7A enzyme has previously been isolated and characterized, and the crystal structure has been determined [21]. MD simulations demonstrated that a single mutation (Tyr371 in *Tre*Cel7A to Ala in *Tha*Cel7A) at the tip of one loop drastically increased the flexibility of an opposing loop across the active site and, thereby, the solvent exposure of the catalytic center [21].

In contrast to distantly related homologs, GH7 CBHs from closely related species have obtained a limited number of evolutionary-driven mutations. The limited set of differences between the enzymes can give important insights to correlate sequence differences with enzyme function and performance. The *Tha*Cel7A and *Tre*Cel7A enzymes can be regarded as naturally occurring variants of *Tat*Cel7A, particularly in terms of the combination of tunnel-enclosing loop motifs. With this view, *Tat*Cel7A is ‘intermediate’ between *Tre*Cel7A and *Tha*Cel7A, combining loop motifs present in either *Tha*Cel7A or *Tre*Cel7A. In this study, we report the biochemical and structural characterization of *Tat*Cel7A. We further compare three cellobiohydrolases, *Tat*Cel7A, *Tha*Cel7A, and *Tre*Cel7A, side-by-side using enzyme activity and performance assays, structural analysis, MD simulation, and reversed conservation analysis (RCA) to correlate differences in sequence with differences in function.

## Results

### Preparation of Cel7A enzymes

The Cel7A enzymes were purified from culture filtrates of *T. atroviride* IOC 4503, *T. harzianum* IOC 3844, and *T. reesei* QM9414 grown in submerged culture under cellulase inducing conditions. Extracellular protein production appeared to be slightly lower for *T. atroviride* than *T. harzianum*, and less protein was obtained than from *T. reesei*. In all cases, Cel7A is the major protein (see Additional file 1: Figure S1; [11, 21]). The yield of purified enzyme per liter of culture was 70 mg for *Tat*Cel7A and 85 mg for *Tha*Cel7A, compared to typical yields of 200–700 mg/L *Tre*Cel7A from *T. reesei* QM9414 [31]. Partial proteolysis with papain [3] could be used to remove the CBM-linker portion from the full-length enzyme and prepare isolated catalytic domains, *Tat*Cel7A_CD, *Tha*Cel7A_CD and *Tre*Cel7A_CD.

### Temperature and pH dependence of enzyme activity and stability

**Fig. 1:**
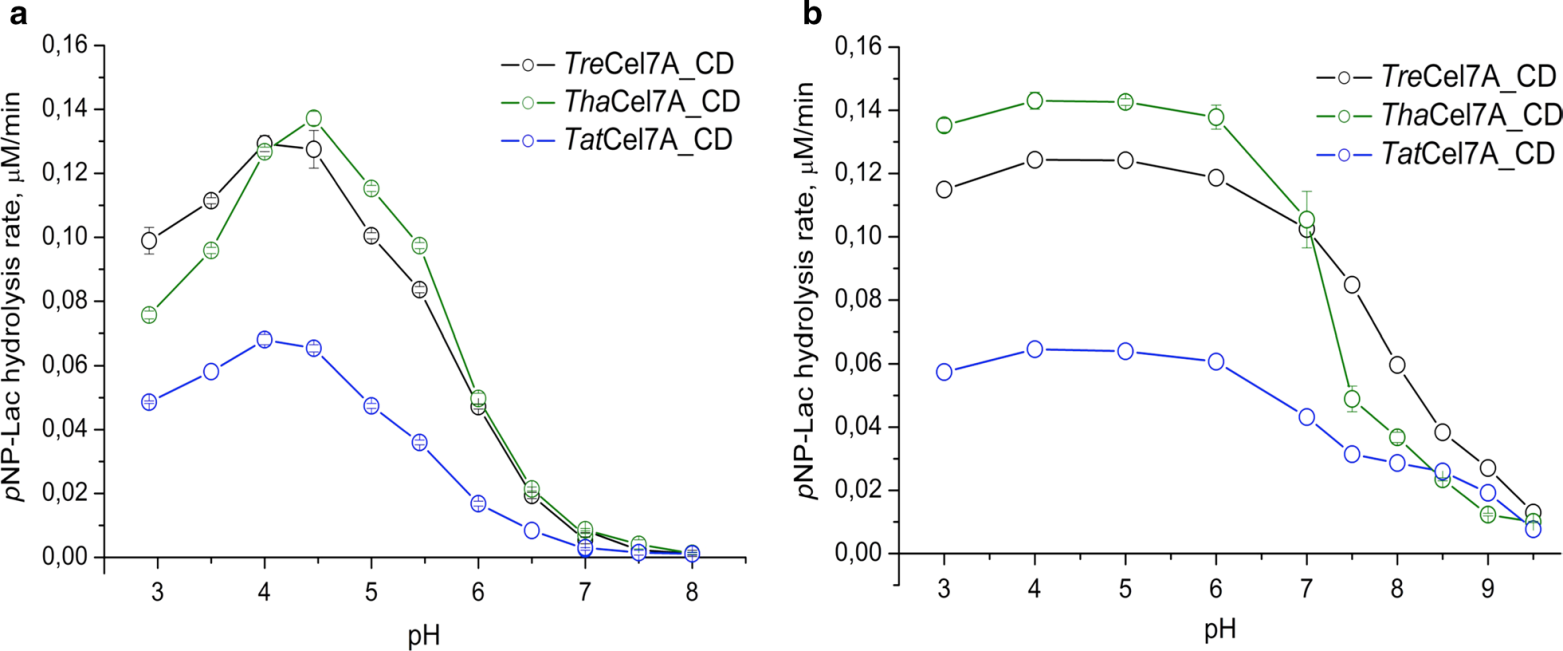
pH dependence for *Tre*Cel7A_CD, *Tha*Cel7A_CD, and *Tat*Cel7A_CD activity on *p*NP-Lac. **a** pH optimum of the activity. Hydrolysis rates were measured in reactions containing 0.15 µM enzyme and 2 mM *p*NP-Lac incubated 30 min at 30 °C at the indicated pHs. **b** pH stability of the enzymes. After enzyme pre-incubation at different pHs for 20 h at 40 °C, residual activities were measured at pH 4.5, 30 °C and 30 min reaction time. Error bars indicate the standard deviation of triplicate measurements

The soluble chromogenic substrate *p*-nitrophenyl-β-lactoside (*p*NP-Lac) was used to monitor and compare the dependence of activity and stability on temperature and pH for the catalytic domains of the Cel7 enzymes. The specific activity of *Tat*Cel7A_CD on *p*NP-Lac is less than half compared to *Tha*Cel7A_CD and *Tre*Cel7A_CD. The pH profiles are rather similar with a pH optimum around pH 4.0–4.5, although *Tha*Cel7A_CD seems to exhibit a slight shift in the alkaline direction and more pronounced drop in activity below pH 4 (Fig. 1a). All three enzymes are essentially inactive from pH 7 and upward. To assess pH dependence of irreversible inactivation, the enzymes were first incubated at different pHs (ranging from pH 3 to 9.5) for 20 h at 40 °C and then assayed at pH 4.5 for residual activity on *p*NP-Lac (Fig. 1b). All three enzymes retained full activity between pH 4 and pH 6 but showed a slight loss of activity at pH 3 and substantial loss from pH 7 and upward. The *Tre*Cel7A_CD seems to be slightly more stable at higher pHs than the other two enzymes. After 20 h incubation at 40 °C and pH 7, the activity dropped to 80, 72, and 52% for *Tre*Cel7A_CD, *Tha*Cel7A_CD, and *Tat*Cel7A_CD, respectively, and at pH 8, to 43, 20, and 24%, respectively. The pronounced sensitivity of *Tha*Cel7A to pH above neutral was observed already during initial attempts of purification. At one stage, the protein was exposed to pH 8, and the activity was lost over time. Consequently, the purification procedures were adapted to avoid exposure of the Cel7A enzymes to pH > 6.

**Fig. 2:**
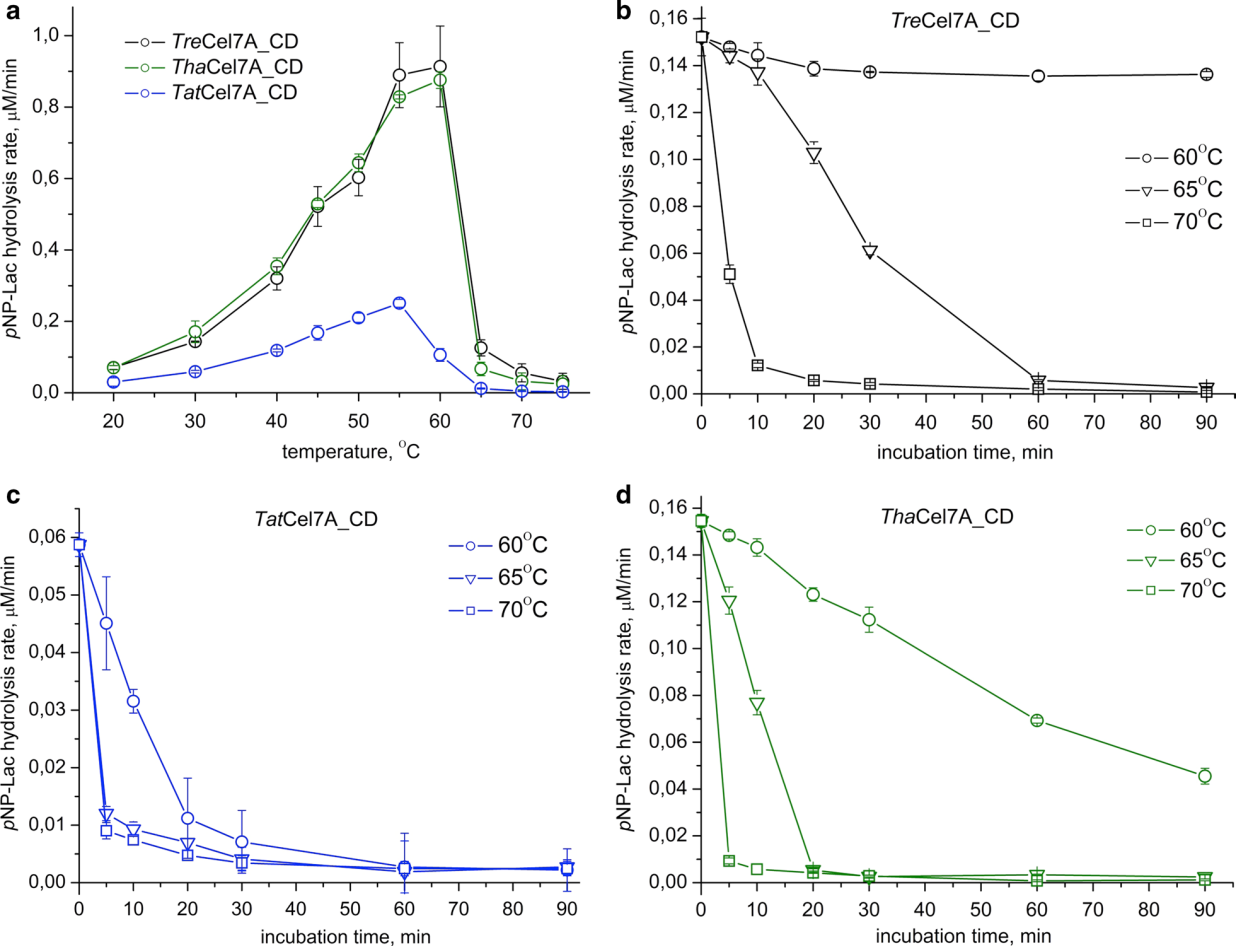
Temperature dependence for *Tat*Cel7A_CD, *Tha*Cel7A_CD, and *Tre*Cel7A_CD activity on *p*NP-Lac. **a** Temperature optimum for 1 h of hydrolysis at indicated temperatures. **b** Temperature stability of *Tre*Cel7A_CD. **c** Temperature stability of *Tat*Cel7A_CD. **d** Temperature stability of *Tha*Cel7A_CD. For **b**–**d** the enzymes were pre-incubated at 60, 65 and 70 °C at pH 4.5. At indicated time points, samples were cooled on ice followed by determination of residual activity at 30 °C. All reactions in **a**–**d** contained 0.15 µM enzyme and 2 mM *p*NP-Lac and were incubated for 1 h at pH 4.5. Thereafter, the increase in *p*NP concentration was measured and divided by the incubation time (60 min) to yield the hydrolysis rates plotted on the *y*-axis. Error bars indicate the standard deviation of triplicate measurements

The temperature dependence plots show that *Tat*Cel7A_CD has a lower optimum temperature, 55 °C, whereas the other enzymes exhibit highest activity at 60 °C (Fig. 2a). The observation that *Tat*Cel7A_CD is most temperature sensitive and *Tre*Cel7A_CD is most thermostable was confirmed by monitoring thermal inactivation over time (Fig. 2b–d). At 60 °C, *Tat*Cel7A_CD is inactivated after 30 min, while *Tha*Cel7A_CD and *Tre*Cel7A_CD retain 30 and 90% activity, respectively, after 90 min. At 70 °C, all three enzymes were inactivated within minutes.

### Enzyme kinetics and cellobiose inhibition

Kinetic properties on *p*NP-Lac and product inhibition by cellobiose was compared for *Tre*Cel7A_CD, *Tat*Cel7A_CD, and *Tha*Cel7A_CD (Table 1). In all cases, Michaelis–Menten kinetics apply, and cellobiose acts as a competitive inhibitor (see Additional file 1: Figure S2). The *K*_M_ values are rather similar for all three, and *k*_cat_/*K*_M_ is practically the same for *Tre*Cel7A_CD and *Tha*Cel7A_CD. However, the catalytic rate constant (*k*_cat_) is significantly lower for *Tat*Cel7A_CD, and its catalytic efficiency (*k*_cat_/*K*_M_) on *p*NP-Lac is only about 25% compared to the others. All three enzymes are highly sensitive to product inhibition, but *Tha*Cel7A_CD and *Tat*Cel7A_CD exhibit somewhat weaker cellobiose binding with 2 and 3 times higher *K*_i_, respectively, than *Tre*Cel7A_CD (Table 1).

**Table 1:** Enzyme kinetics parameters with *p*NP-Lac as substrate and inhibition constants for cellobiose, at pH 4.5 and 30 °C

Enzyme	*k*_cat_ (s^−1^)	*K*_M_ (mM)	*k*_cat_/*K*_M_ (s^−1^ M^−1^)	*K*_i_ (µM)
*Tre*Cel7A_CD	0.057	0.72	79	24
*Tat*Cel7A_CD	0.019	1.00	19	72
*Tha*Cel7A_CD	0.067	0.90	75	49

*K*_i_ is the competitive inhibition constant with 100 µM cellobiose in the reactions. The RMSD between calculated and experimental reaction rates was 3.4, 4.0, and 2.4% for *Tre*Cel7A_CD, *Tat*Cel7A_CD, and *Tha*Cel7A_CD, respectively

### Initial cellulose hydrolysis

**Fig. 3:**
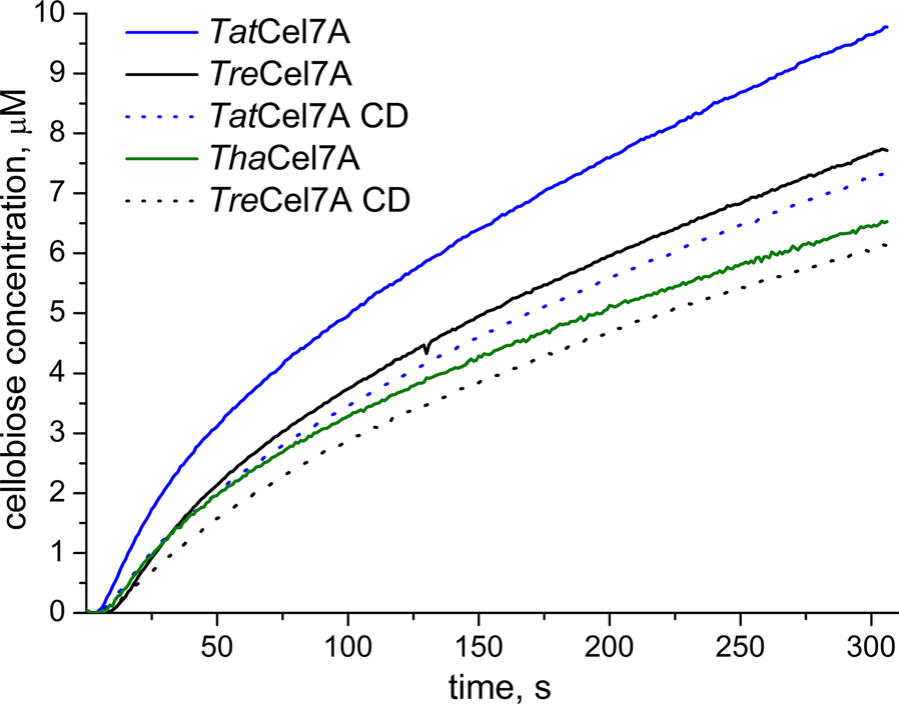
Real-time progress curves of cellulose hydrolysis. Cellobiose production during initial hydrolysis of BMCC by *Tre*Cel7A, *Tre*Cel7A_CD, *Tat*Cel7A, *Tat*Cel7A_CD, and *Tha*Cel7A enzymes was monitored with an amperometric CDH enzyme biosensor. The reactions were carried out at 25 °C, pH 5.0, with 3.3 g/L of BMCC and 50 nM enzyme. Each enzyme kinetic curve was measured in duplicate, and the average curve is plotted for each enzyme. For more details, see Additional file 1: Figure S3

The initial production of cellobiose from bacterial microcrystalline cellulose (BMCC) was monitored in real time using an amperometric cellobiose dehydrogenase (CDH) enzyme biosensor [32, 33]. Figure 3 shows the progress curves for full-length *Tat*Cel7A, *Tre*Cel7A, and *Tha*Cel7A and the *Tat*Cel7A_CD and *Tre*Cel7A_CD catalytic domains.

The experimental data in Fig. 3 were fit to a processive model [33, 34]. The model consists of three distinct steps: enzyme–substrate association, processive catalysis, and enzyme dissociation, governed by the rate constants *k*_on_, *k*_cat_, and *k*_off_, respectively. Further, the model contains an apparent processivity parameter, *n*, which represents the average number of sequential catalytic cycles. For further details, refer to Additional file 1. The kinetic parameters derived from non-linear regression are given in Table 2. The apparent processivity of all the analyzed enzymes was similar; however, *Tat*Cel7A exhibited 20% higher apparent processivity than *Tha*Cel7A and 9% higher than *Tre*Cel7A. The catalytic domains have approximately the same processivity number as the corresponding full-length variants. The main difference in function between the full length enzymes (*Tre*Cel7A and *Tat*Cel7A) and catalytic domains (*Tre*Cel7A_CD and *Tat*Cel7A_CD) manifested in *k*_on_, which was significantly lower for each of the catalytic domains. When comparing full-length enzymes, the kinetic parameters were similar, except for a significantly higher *k*_cat_ for *Tat*Cel7A. Since all the enzymes were purified from the native host, a test of endoglucanase (EG) activity in the CBH samples was performed, using AZCL-HE-cellulose (Megazyme) as substrate. The estimated amount of EG was negligible and was not considered to affect the kinetic parameters.

**Table 2:** Kinetic parameters derived from progress curves of initial BMCC hydrolysis: association (*k*_on_), catalytic (*k*_cat_), and dissociation (*k*_off_) rate constants, and apparent processivity number (*n*)

Enzyme	*k*_on_ (g^−1^ L s^−1^)	*k*_cat_ (s^−1^)	*k*_off_ (s^−1^)	*n*
*Tre*Cel7A	0.0055 ± 0.0002	4 ± 0.2	0.0066 ± 0.0005	89 ± 5
*Tre*Cel7A_CD	0.0034 ± 0.0005	4.9 ± 0.7	0.0049 ± 0.0012	88 ± 5
*Tha*Cel7A	0.0056 ± 0.0002	4.8 ± 0.4	0.0061 ± 0.0002	74 ± 1
*Tat*Cel7A	0.0071 ± 0.0001	8.3 ± 0.3	0.0071 ± 0.0001	97 ± 1
*Tat*Cel7A_CD	0.0044 ± 0	6.8 ± 0.3	0.0066 ± 0.0002	87 ± 3

These parameters were derived for the 0–200 s pre-steady-state time interval. The substrate load was 3.3 g/L, and the enzyme concentration was 50 nM

### Enzyme performance on pretreated biomass

**Fig. 4:**
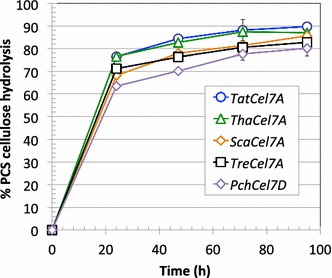
Performance assay on pretreated corn stover (PCS). Synergistic conversion of PCS (5.0 g glucan/L) to soluble sugar at 40 °C and pH 5.0 was monitored using full-length GH7 CBH enzymes (~ 2.5 µM), together with a GH7 endoglucanase and a β-glucosidase (28, 1.9, and 0.5 mg enzyme per gram glucan, respectively). In addition to the three *Trichoderma* CBHs, Cel7A from *Scytalidium* sp. (*Scy*Cel7A; identical to the enzyme called *Geotrichum candidum* Cel7A in [3]) and Cel7D from *Phanerochaete chrysosporium* were also analyzed at the same time; the results are shown for comparison. Experiments were performed in duplicate

The efficiency of the Cel7A enzymes in synergistic lignocellulose saccharification was assessed by performance assays on dilute acid-pretreated corn stover (PCS) as substrate. Full-length GH7 CBHs, together with a GH7 endoglucanase (*Trichoderma longibrachiatum* Cel7B/EG I) and a β-glucosidase, were incubated with PCS at pH 5 and 40 °C, and the release of soluble sugar was followed for 96 h (Fig. 4). The highest conversion was obtained with *Tat*Cel7A, closely followed by *Tha*Cel7A, both of which appeared more efficient than *Tre*Cel7A. The conversion after 47 h was 84, 83, and 76% for *Tat*Cel7A, *Tha*Cel7A, and *Tre*Cel7A, respectively.

### Sequence comparison of *Trichoderma* spp. Cel7A orthologs

**Fig. 5:**
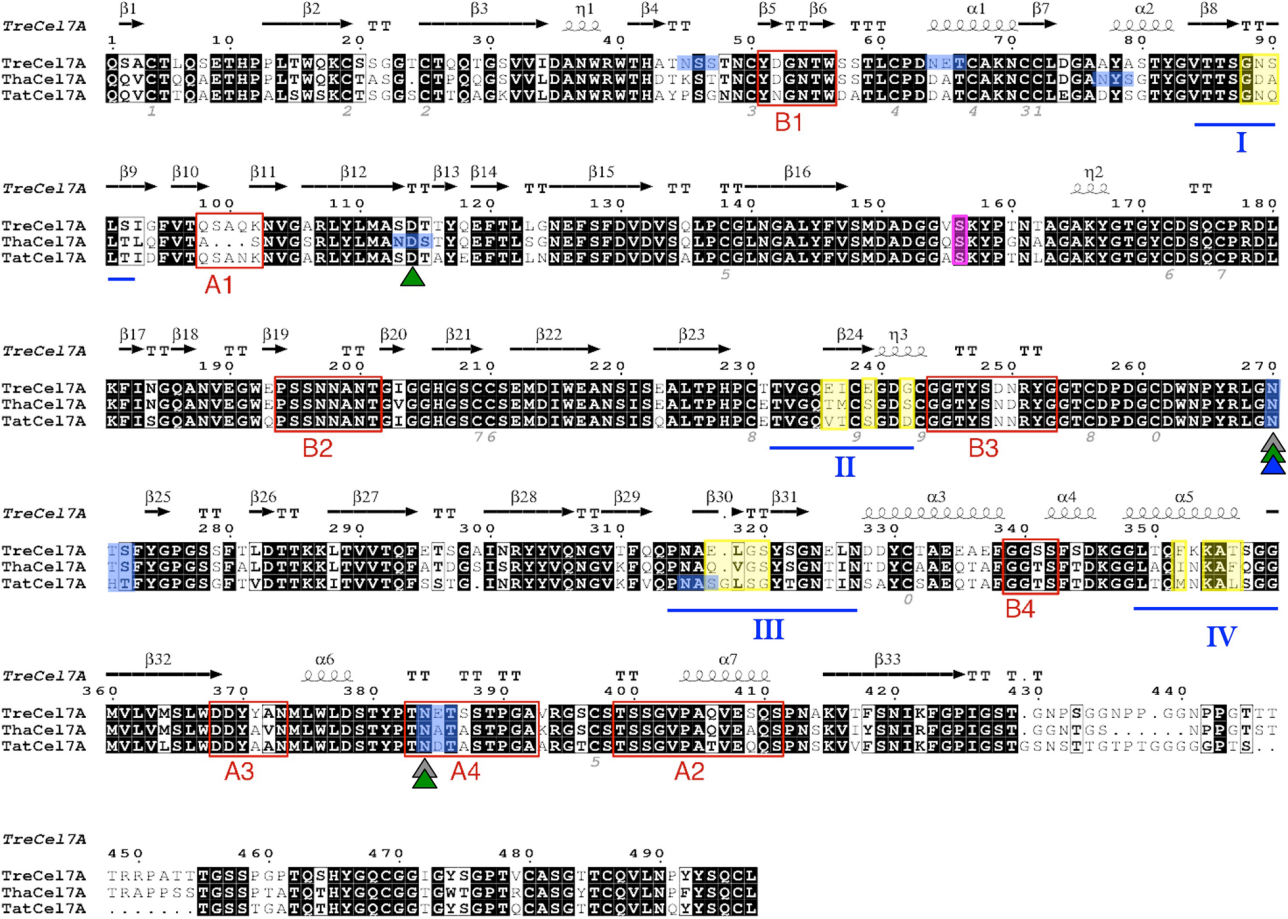
Alignment of the GH7 *Tre*Cel7A, *Tha*Cel7A, and *Tat*Cel7A protein sequences. Secondary structural elements of the *Tre*Cel7A structure are indicated above the alignment (β-strand arrows and α-helices) based on the 3D structure of the catalytic domain (1CEL). Strictly identical residues are marked in white letters on a black background. Regions of conserved, highly similar residues are framed in thin-lined boxes with bold letters. The figure was prepared using the ESPript web server with default parameters (http://espript.ibcp.fr; [82]). Red frames indicate loop regions of interest, with loop nomenclature underneath. The regions highlighted in blue denote N-glycosylation motifs; the colored triangles indicate the *N*-glycosylated asparagine residues observed in structures, where gray triangles correspond to *Tre*Cel7A, green triangles to *Tha*Cel7A, and the blue triangle to *Tat*Cel7A. Sections of interest defined by RCA (reverse conservation analysis) are marked with blue lines and corresponding identifiers (I–IV), and residues with high *S* scores are marked in yellow

The protein sequences of *Tat*Cel7A, *Tre*Cel7A, and *Tha*Cel7A all contain a signal peptide for secretion pathway targeting in their N-termini, followed by a GH7 catalytic domain and a C-terminal, fungal-type cellulose-binding module (CBM1). The linker region is significantly shorter in *Tat*Cel7A (22 residues), containing mostly glycine residues, whereas *Tre*Cel7A has the longest linker (30 residues) of the three enzymes (Fig. 5). A structure-based sequence alignment (excluding the signal peptide) confirms that the sequence identity is high (~ 80%; Fig. 5). All previously described loop regions are conserved in length [20], with the exception of loop A1, where three residues at the tip of the loop are missing in *Tha*Cel7A compared to *Tre*Cel7A and *Tat*Cel7A. In addition, there are three more indels in the catalytic domains, represented by one residue deletion (between Gly298 and Ile299) and one residue insertion (Gly317) in *Tat*Cel7A, and one residue deletion in *Tha*Cel7A (Ser24 in *Tat*Cel7A). Two N-glycosylation sites are conserved in all three enzymes (i.e., Asn270 and Asn384 in *Tat*Cel7A).

### Crystal structures of *Tat*Cel7A_CD

The *Tat*Cel7A_CD protein was successfully crystallized, and two structures were solved, one apo structure without sugars bound (APO) and one thio-cellotrioside complex (SG3). Both structures were refined at 1.7 Å resolution in space group *P*21 with two protein chains, A and B, in the asymmetric unit. X-ray diffraction data and structure refinement statistics are summarized in Table 3.

**Table 3:** X-ray diffraction data and refinement statistics for the *Tat*Cel7A_CD structures

	Apo structure (APO)	Thio-cellotriose complex (SG3)
Data collection		
PDB accession no.	5O5D	5O59
Beam line	I911-3, MAX-lab	ID23-1, ESRF
Space group Cell dimensions	P21	P21
a, b, c (Å)	56.17, 71.67, 104.22	55.85, 71.34, 102.91
Wavelength (Å)	1.0	1.0
Resolution (Å)^a^	34.74–1.72 (1.82–1.72)	58.63–1.75 (1.85–1.75)
Unique reflections	87,266	67,271
Multiplicity	5.2 (4.6)	2.8 (2.9)
Completeness (%)	99.9 (99.6)	83.5 (79.1)
*I*/*σI*	9.2 (3.4)	8.8 (2.3)
R merge (%)^b^	13.2 (44.0)	8.7 (50.4)
Refinement		
*R*_work_/*R*_free_ (%)	14.8/17.3	15.6/19.0
Protein atoms: no., average B-factor (Å^2^)	6571 17.9	6531 18.1
RMSD Bond angle (°)	1.052	1.353
RMSD Bond length (Å)	0.0051	0.008

^a^Data within parentheses are for the outermost resolution shell

^b^*R*_merge_ = $\sum_{h} \sum_{\text{i}} {{\left| {I\left( h \right)_{i} - \left\langle {I\left( h \right)} \right\rangle } \right|} \mathord{\left/ {\vphantom {{\left| {I\left( h \right)_{i} - \left\langle {I\left( h \right)} \right\rangle } \right|} {\sum_{h} \sum_{i} I\left( h \right)_{i} }}} \right. \kern-0pt} {\sum_{h} \sum_{i} I\left( h \right)_{i} }}$ where *I*(*h*)_*i*_ is the intensity of reflection *h* and $\left\langle {I\left( h \right)} \right\rangle$ is the average value over multiple measurements

**Fig. 6:**
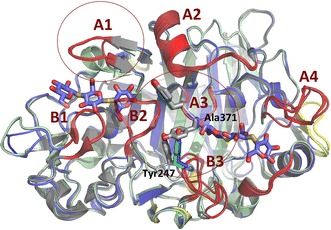
Overall structure of *Tat*Cel7A_CD. The structure of the *Tat*Cel7A_CD thio-cellotrioside ligand complex (blue) is superposed with *Tha*Cel7A_CD (green; PDB code 2YOK) and *Tre*Cel7A_CD (gray; PDB code 4C4C). The thio-cellotrioside ligands are colored with slate blue carbon atoms. The cellononaose ligand of the *Tre*Cel7A structure is not shown. Loop regions are highlighted and labeled in red. Three naturally occurring variants of loop A1 and A3/B3 loop interactions are highlighted with red circles. Amino acid residues involved in A3/B3 loop contacts are shown in sticks and colored for *Tat*Cel7A, *Tha*Cel7A, and *Tre*Cel7A accordingly

**Fig. 7:**
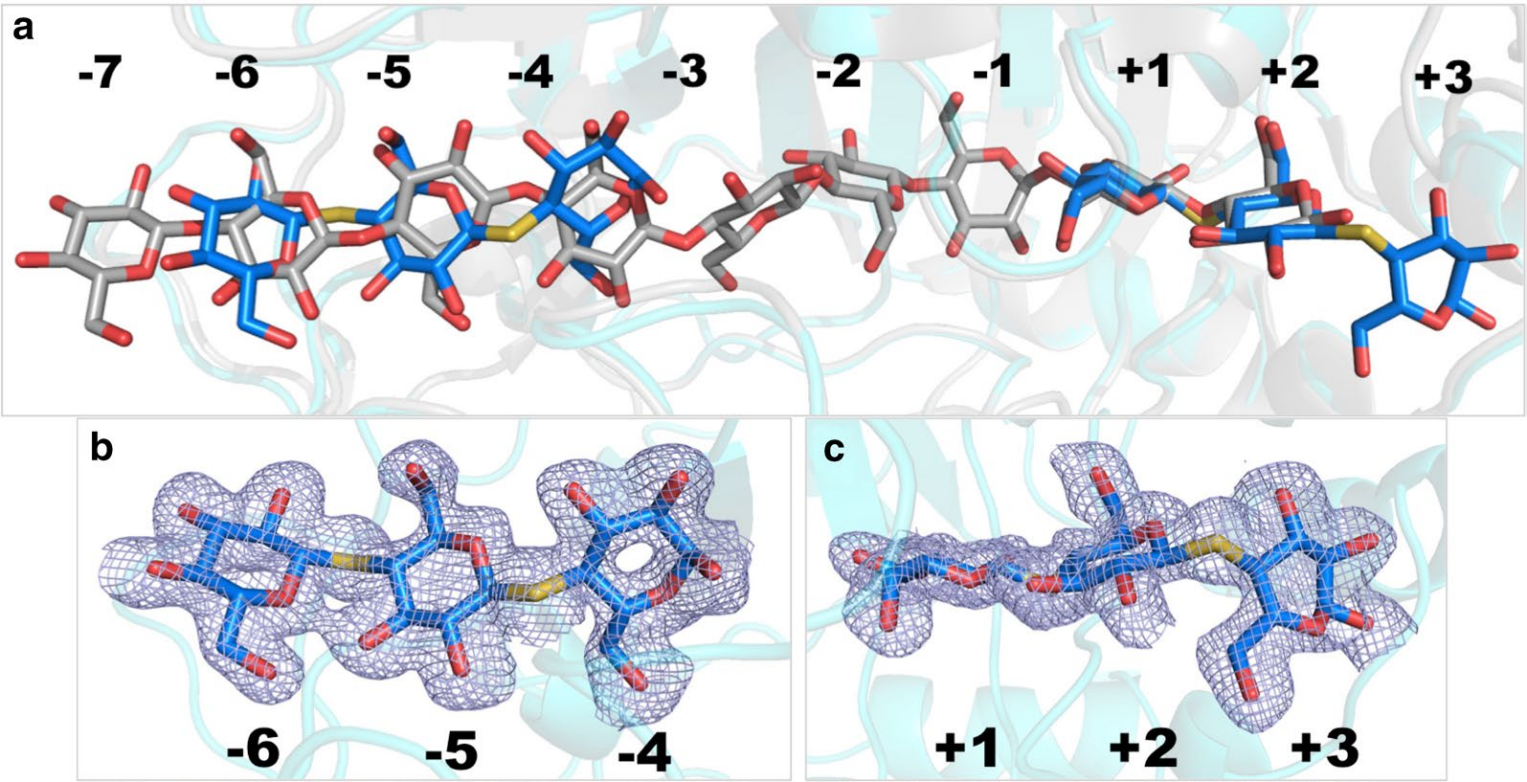
Glycan binding in the *Tat*Cel7A_CD thio-cellotrioside complex structure. **a** Superposition of ligand binding in the SG3 structure (blue) with cellononaose in the *Tre*Cel7A Michaelis complex (4C4C; gray). **b** Electron density for the ligand at subsites − 6/− 5/− 4. **c** Electron density for the ligand at the + 1/+ 2/+ 3 position. The 2*F*_o_–*F*_c_ electron density maps are contoured at 0.26 e/A^3^

The APO structure was obtained from co-crystallization of *Tat*Cel7A_CD with thio-linked cellobiose, but no cellobiose is seen in the structure. In the SG3 structure, from co-crystallization with thio-linked cellotriose, there are two cellotrioside molecules bound to each protein chain, in subsites − 6/− 5/− 4 and + 1/+ 2/+ 3, respectively (Figs. 6, 7). In both protein chains of both structures, all amino acids from 1 to 430 of *Tat*Cel7A_CD could be included in the structure model. The N-terminal glutamine residue is cyclized to pyroglutamate (PCA1), the C-terminal residue is Gly430, and all the 20 cysteines form disulfide bridges. N-glycosylation is visible at one site, with one GlcNAc residue attached to Asn270. In the APO structure, there is distinct density for a Bis–Tris molecule bound to the catalytic residues, Glu212, Asp214, and Glu271, at the catalytic center of the active site, whereas glycerol is found in a similar position in the SG3 structure.

**Fig. 8:**
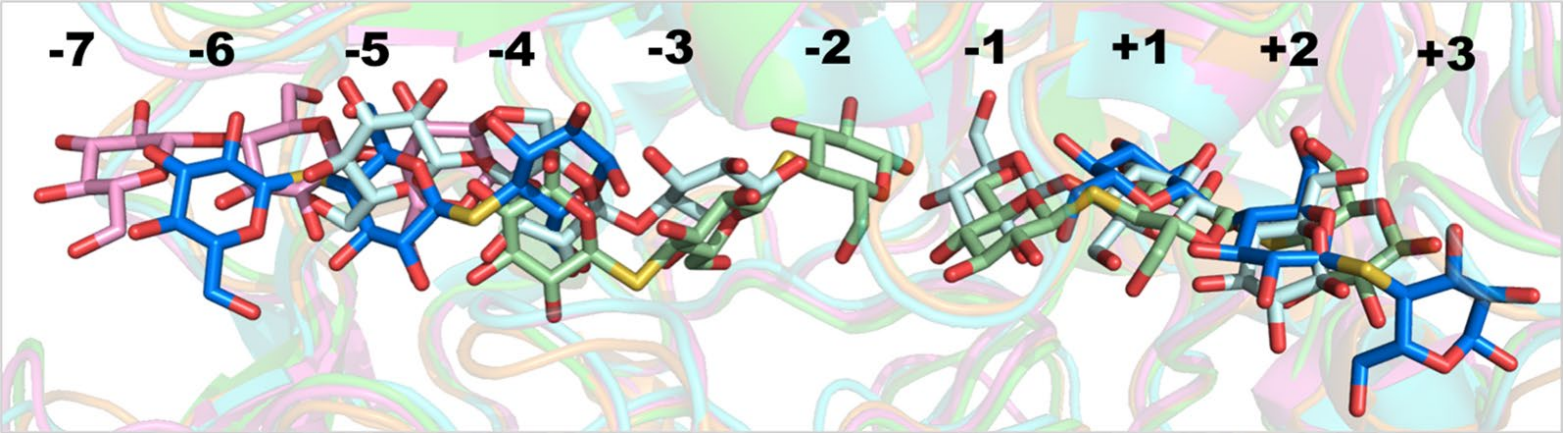
Superposition of ligands from Cel7/cellotrioside complex structures. The following colors are used: pale cyan, *Melanocarpus albomyces* Cel7B (2RFZ, chain A); pink, *Limnoria quadripunctata* Cel7B (4HAQ, chain B); pale green, Cel7A from *Scytalidium* sp. (*Scy*Cel7A; identical to the enzyme called *G. candidum* Cel7A in Borisova et al. [3]) (4ZZT); slate blue, *Tat*Cel7A. Yellow bonds indicate the 1,4-S-linkage in thio-cellotrioside ligands (from *Scy*Cel7A and *Tat*Cel7A)

The position and conformation of the thio-cellotrioside ligands in the SG3 structure are well defined by the electron density, as shown in Fig. 7. For the ligand in the − 6/− 5/− 4 position at the tunnel entrance, all glucose residues adopt the ^4^*C*_1_ chair conformation, but the C1 hydroxyl at the reducing end in subsite − 4 is predominantly in the α-position, with very weak density for a β-hydroxyl. Interestingly, the sugar ring at each site is flipped upside down compared to the orientation in the Michaelis complex of *Tre*Cel7A (Fig. 7a). The binding may represent a sliding intermediate during processive cellulose hydrolysis. The flipped orientation could also be a consequence of the slight difference in geometry of the thio-ether linkage compared to that of a standard *O*-glycosidic bond. On the other hand, another Cel7A structure (4ZZT) shows two thio-cellotrioside molecules bound in subsites − 4/− 3/− 2 and − 1/+ 1/+ 2 in the normal orientation (Fig. 8). The other thio-cellotrioside molecule in the SG3 structure, at + 1/+ 2/+ 3, is in register and aligns well with the glycan binding at the product sites of the *Tre*Cel7A Michaelis complex. The + 1 and + 2 glucosides are in ^4^*C*_1_ chair conformations, whereas the + 3 unit at the reducing end adopts a ^1^*S*_3_ skew conformation, again with α-hydroxyl predominance at the anomeric carbon. The sugar ring distortion is not induced by crystal contacts, since there are no interactions with any neighbor protein in this region.

**Fig. 9:**
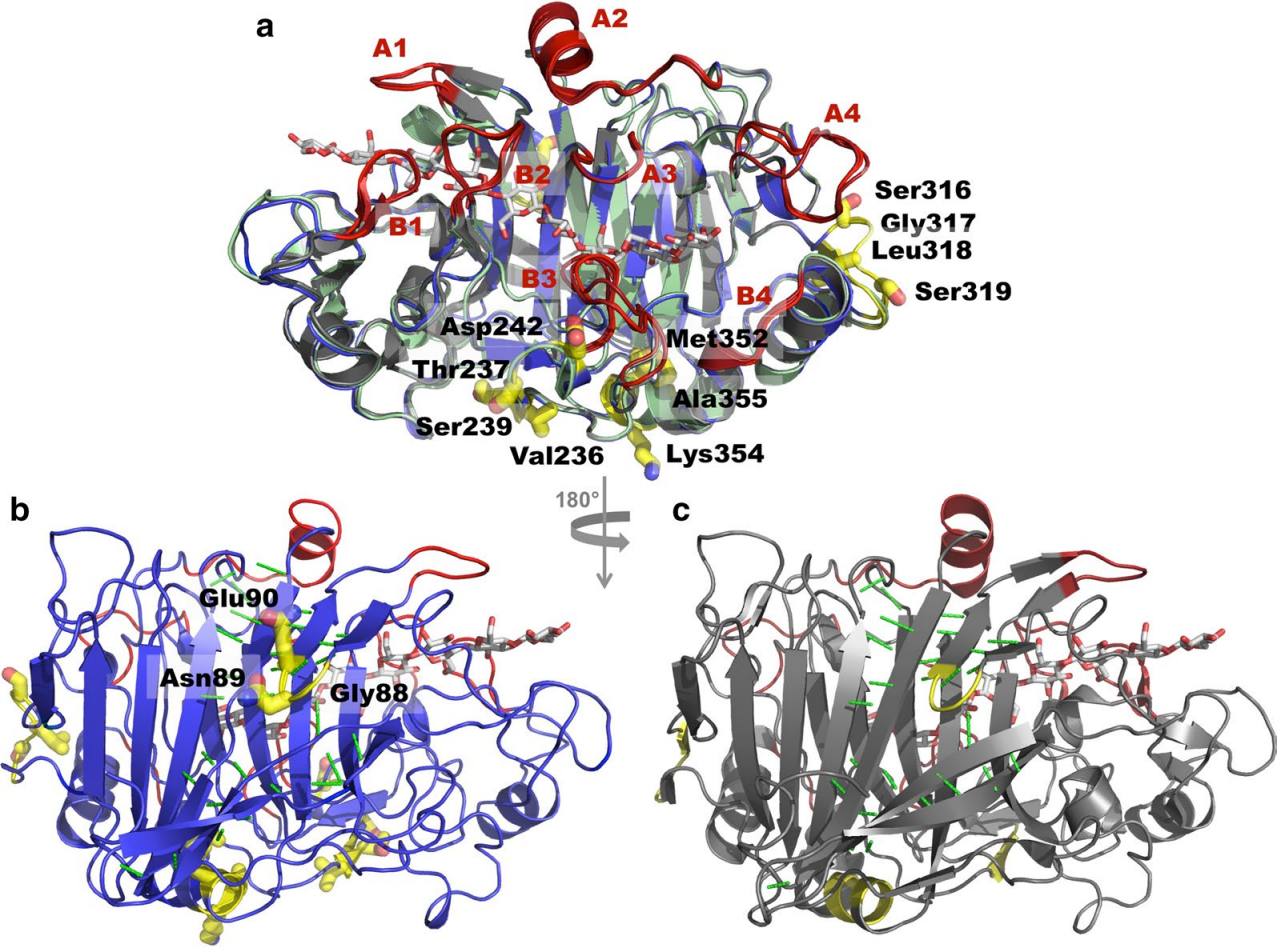
Comparison of *Trichoderma* spp. Cel7A structures. **a** Superposition of *Tat*Cel7A_CD APO (blue), *Tha*Cel7A_CD (green; PDB code 2YOK), and *Tre*Cel7A_CD (gray; PDB code 4C4C). Loop regions are highlighted in red, and cellononaose from 4C4C is shown with white carbon atoms. Sections I–IV, defined by RCA, are marked in yellow, and amino acid residues with high *S*-scores in *Tat*Cel7A are shown as sticks. **b** Back side of *Tat*Cel7A_CD APO structure. **c** Back side of *Tre*Cel7A_CD. In **b**, **c**, polar interactions between β-strands are shown as green dashed lines

Overall, the *Tat*Cel7A_CD structures are very similar (0.18 Å root mean square deviation, RMSD), although there is a general ‘tightening’ of the protein around the active site in SG3 compared to APO, which is most pronounced at the A4-loop near the product sites (Fig. 9). The fold is very similar to that of *Tre*Cel7A_CD and *Tha*Cel7A_CD (RMSD 0.54 and 0.44 Å, respectively), as expected from the high sequence identity (80 and 82%, respectively).

The lining of the cellulose-binding path is identical in the three enzymes except at two locations, loop A1 at the entrance to the tunnel and loop A3 near the catalytic center (Fig. 6). In loop A1, Glu101 in *Tre*Cel7A binds the 6-hydroxyl of the glucose unit in subsite − 6. This residue is replaced by a shorter sidechain, Asn101, in *Tat*Cel7A. Nevertheless, Asn101 may still bind to the cellulose chain but probably results in a weaker interaction. In *Tha*Cel7A, a corresponding interaction is completely absent, since the tip of the A1 loop is three residues shorter (99–101 in *Tat*Cel7A and *Tre*Cel7A) [21]. The shorter A1 loop does not reach over subsite − 6, making the entrance to the tunnel more open in *Tha*Cel7A (Fig. 6). At the second location, loop A3, Tyr371 in *Tre*Cel7A interacts with Tyr247 at the tip of the opposing B3 loop. Tyr371 is replaced by an alanine in both *Tat*Cel7A and *Tha*Cel7A, and there are no direct interactions between loops A3 and B3 across the tunnel. Also, loop B3 has moved outwards in *Tat*Cel7A and *Tha*Cel7A compared to the *Tre*Cel7A structure (5.5 Å distance between Tyr247 OH of *Tat*Cel7A and *Tre*Cel7A). At the next position in loop A3, Ala372 of *Tat*Cel7A and *Tre*Cel7A is replaced by a valine in *Tha*Cel7A, which appears to influence the dynamics of the adjacent A4 loop near the product site.

The overall backbone structures of the three enzymes are very similar, apart from small deviations in loops and turns at the surface of the protein. However, there is one small, but significant, difference in *Tat*Cel7A that locally affects protein folding. A one-residue insertion, Gly317, is present in the region 316–319 (section III in the sequence alignment, Figs. 5, 9), where there is a β-strand-turn in *Tre*Cel7A and *Tha*Cel7A, which supports the A4 loop at the product end of the active site. Also, a glutamine (*Tre*Cel7A) or glutamic acid (*Tha*Cel7A) is substituted by Ser316 in *Tat*Cel7A, thus introducing a shorter side chain. This substitution, followed by the glycine insertion, disrupts β-strand interactions, and the 315–317 residues bulge outwards in *Tat*Cel7A. Furthermore, Ser316 introduces an N-glycosylation motif (at Asn314) not present in the two other enzymes (Fig. 5). This glycosylation site is close in space to the Asn270 N-glycosylation. Although glycans potentially attached to Asn314 could not be observed in the *Tat*Cel7A structures, a glycan present at this site would be in direct contact with the one at Asn270.

Overall, *Tat*Cel7A appears to have fewer secondary structure interactions compared to *Tre*Cel7A and *Tha*Cel7A, with shorter β-strands and α-helixes at several locations (see overlay of the structures in Fig. 9). This suggestion is corroborated by a lower number of total native contacts found from the MD simulations (see below).

### Molecular dynamics (MD)

**Fig. 10:**
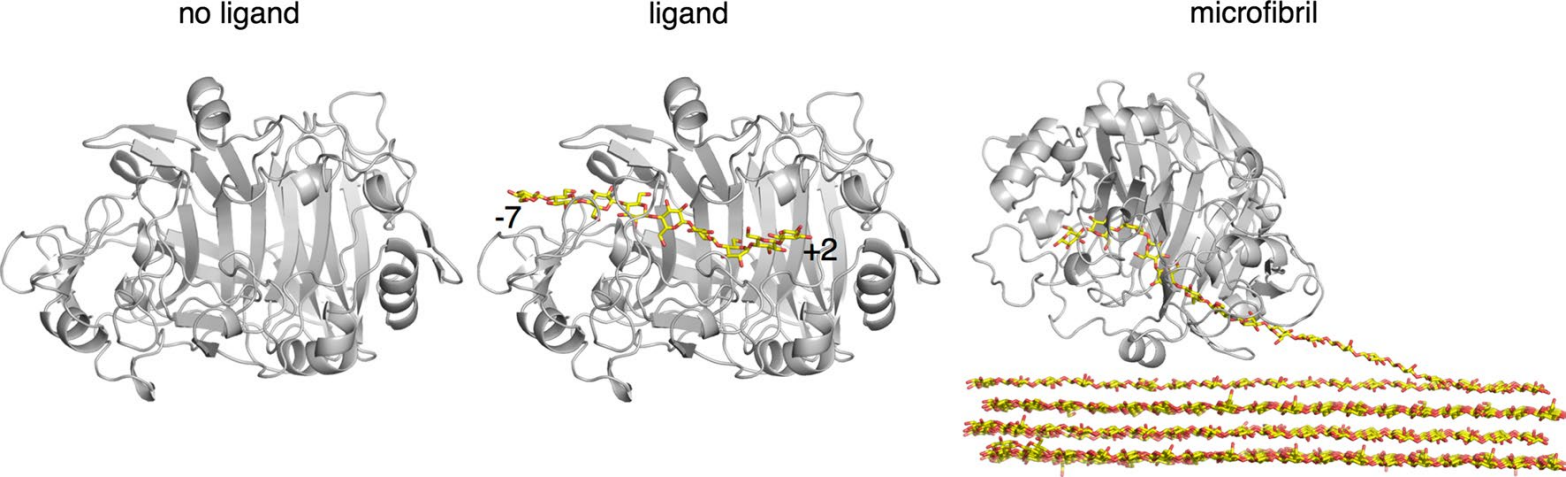
Illustration of cellulose active site occupancies examined using MD simulation. Three simulation cases were conducted for each of the three cellulose catalytic domains, including the ligand-free state (no ligand), the cellononaose-bound state (ligand), and the cellulose Iβ microfibril-bound state (microfibril). Each simulation was conducted in the *NVT* ensemble at 300 K for 100 ns using explicit solvent (not shown for clarity). The catalytic domain of the protein is shown in gray cartoon. Cellononaose and the cellulose microfibril are shown in red and yellow stick

We performed MD simulations to understand how structural differences in *Tat*Cel7A_CD, *Tre*Cel7A_CD, and *Tha*Cel7A_CD manifest in protein dynamics. We also examined the effect of bound substrate on protein dynamics for each of the three cellulases, conducting simulations in the apo state and bound to both cellononaose and crystalline cellulose substrate (Fig. 10).

**Fig. 11:**
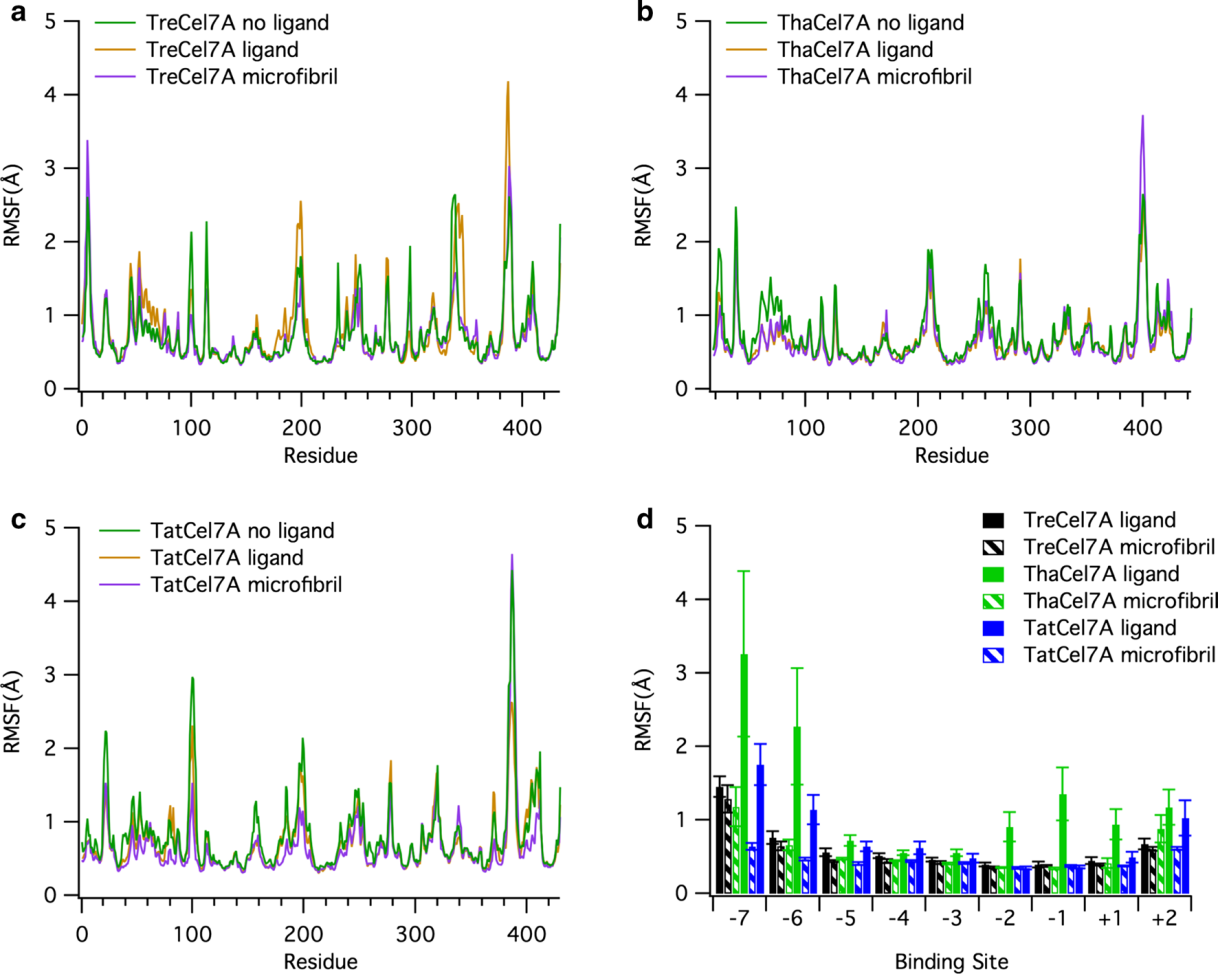
RMSF from molecular dynamics simulations. The RMSF of the **a** *Tre*Cel7A, **b** *Tha*Cel7A, and **c** *Tat*Cel7A backbones and **d** the ligand and microfibril over 100-ns simulations at 300 K. Error bars shown in **d** were obtained through 2.5 ns block averaging. The molecular simulation data for *Tre*Cel7A was reported previously and is shown here for comparison [3]

**Fig. 12:**
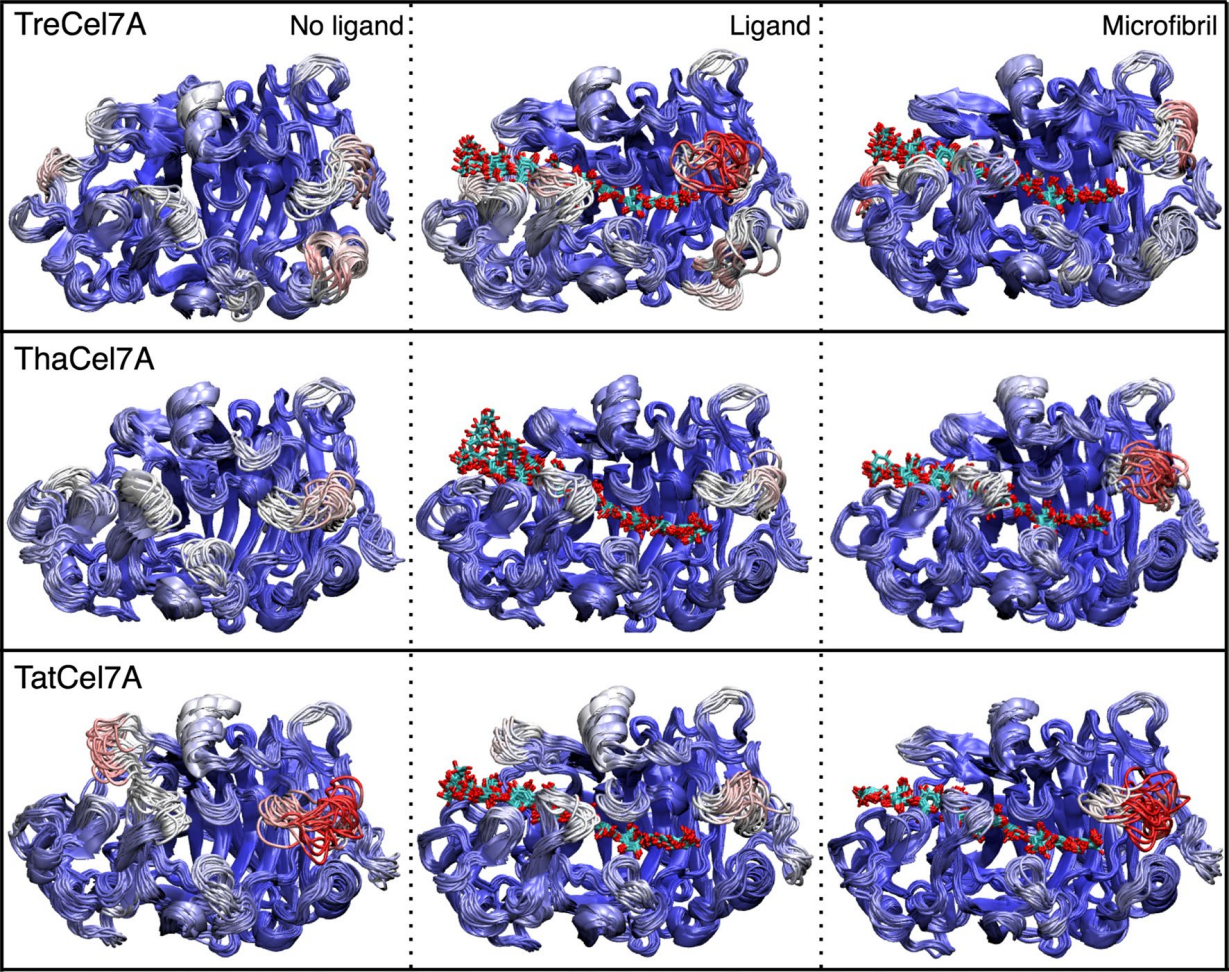
RMSF comparison of the protein backbone at 300 K. The backbone is colored by a gradient from blue to white to red, representing lower to higher fluctuations. Red regions indicate larger fluctuations (RMSF > 4 Å). The cellooligosaccharide chain is colored by atom; oxygen is red, and carbon is cyan

The root mean square fluctuations (RMSF) of the protein backbone are similar for the three proteins, with elevated RMSF values in the loop regions. The fluctuations are largest around loop A4 (residues 390–410, Figs. 11, 12). *Tat*Cel7A is stabilized when complexed with the cellulose microfibril, which is indicated by a decrease in overall fluctuations. The decrease in overall fluctuation is much less pronounced in the other two enzymes (Figs. 11, 12). Inside the binding tunnel, at subsites − 5 to − 2, the cellononaose ligand and the chain of the microfibril fluctuate very little in either of the three cellulases; however, towards the ends of the active site, subsites − 7/− 6 and + 1/+ 2, the ligands naturally fluctuate more, as the ligands are more solvent exposed (Fig. 11d). Higher RMSF in *Tha*Cel7A subsites − 7/− 6 correlate well with the shorter A1 loop, which makes the entrance to the tunnel wider in this enzyme. Interestingly, this difference is only seen in the simulations with the cellononaose ligand. When complexed with the cellulose microfibril, the ligand fluctuations are nearly the same for the three enzymes along the length of the active site. Throughout all of the MD simulations, either in presence of the crystalline cellulose or while complexed with the cellononaose oligomer in solution, the reducing end of the ligand (+ 2 site) in the active site remained in the β-anomeric configuration. This configuration arises as a requirement of the implemented carbohydrate force field used for all of our MD simulations, which restricts the + 2 pyranose to the β-anomeric configuration by virtue of spring constants on the angle and dihedral parameters. While it is feasible that the pyranose rings could temporarily occupy an α-anomeric configuration, the energy barrier to do so is quite large.

**Fig. 13:**
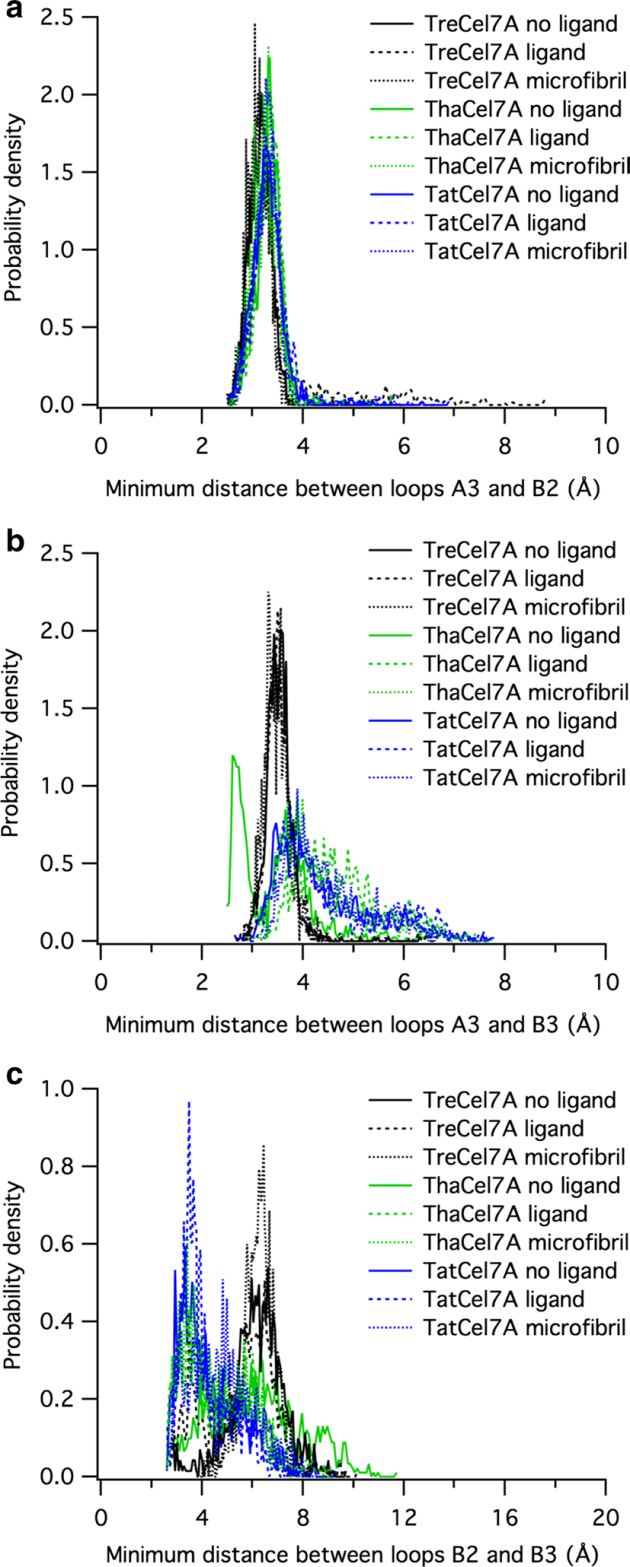
Active site loop distances from molecular simulation. Histograms of the minimum distances between **a** loops A3 and B2, **b** loops A3 and B3, and **c** loops B2 and B3, evaluated from 100-ns simulation trajectories of *Tre*Cel7A, *Tha*Cel7A, and *Tat*Cel7A

Despite high sequence and structural similarity, MD simulations reveal distinct differences in terms of loop dynamics between *Tat*Cel7A, *Tha*Cel7A, and *Tre*Cel7A, illustrated by histograms of distances between tunnel-enclosing loops, A3–B2, A3–B3, and B2–B3, respectively, over the course of simulation (Fig. 13). Most notably, the active site loops of *Tre*Cel7A appear to be significantly more stationary than either *Tha*Cel7A or *Tat*Cel7A. One conformational state is strongly preferred, as demonstrated by small fluctuations (within 1–2 Å) of the inter-loop distances around a single peak. Both the A3/B2 loops and the A3/B3 loops, ‘A’ and ‘B’ designating opposite sides of the active site, remain in direct contact during most of the simulation. In this configuration, the tunnel is physically closed, in the sense that a cellulose chain would only be able to enter or exit through either end of the active site and not “sideways”. Occasionally, the loops do separate enough (> 6 Å) to allow a cellulose chain to pass (Fig. 13a).

The *Tat*Cel7A and *Tha*Cel7A enzymes behave similar to *Tre*Cel7A with respect to the A3/B2 loop distances, exhibiting small fluctuations around a single peak. However, the A3/B3 and B2/B3 loop distances are much more variable than in *Tre*Cel7A, likely due to the increased flexibility of loop B3 over B2. In *Tat*Cel7A the A3/B3 loop distance was most frequently between 3.5 and 4 Å, though the distance could also hover between 5 and 7 Å, indicating that loop B3 moves smoothly between a closed and an open conformation, without any major energy barrier (Fig. 13b). The B3 loop behavior is similar regardless of the presence or absence of ligand/microfibril in the active site, which is a contrast to the B3 loop behavior in *Tre*Cel7A where the loop is most often in a closed conformation. Yet another behavior was observed in *Tha*Cel7A, where the B3 loop exhibits a bimodal distribution in the absence of ligand; the B3 loop seemingly flips between two closed conformations. The A3/B3 distance is very short for the primary conformation. With a bound ligand or microfibril, there is no evidence of the short-distance state, and loop B3 appears to fluctuate over a larger range of more open conformations.

**Fig. 14:**
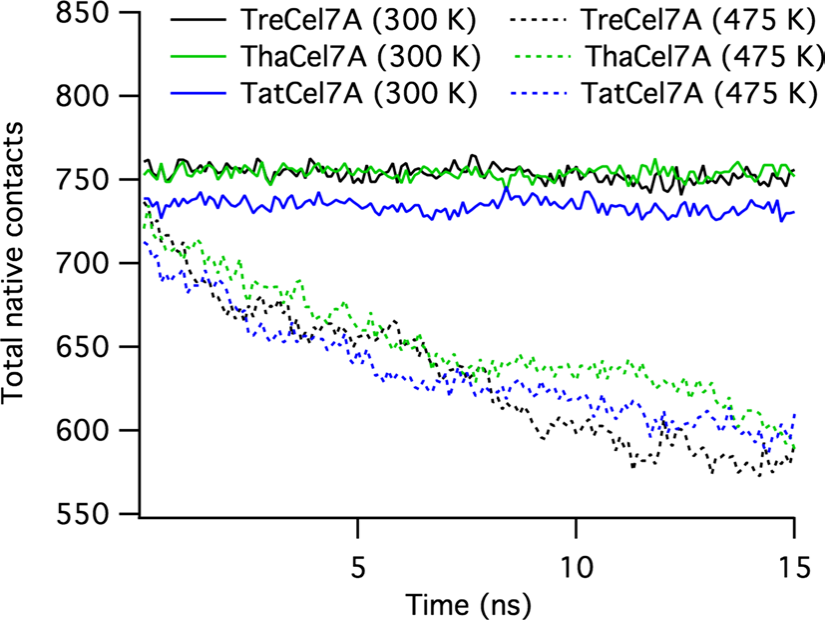
Total number of native contacts formed by *Tre*Cel7A, *Tat*Cel7A, and *Tha*Cel7A at 300 and 475 K. The total number of native contacts was determined as an average of three independent MD simulations at two temperatures, 300 K (solid lines) and 475 K (dashed lines). The high temperature simulations were performed for 15 ns, whereas the triplicate 300 K simulations were conducted for 50 ns; only 15 ns of the 300 K trajectories are shown here for comparison. In each case, the simulation was conducted without a ligand, in explicit solvent. To determine the total number of native contacts of each trajectory, the number of native contacts formed by each residue was first evaluated. Here, a native contact was defined as any amino acid whose side chain center of geometry was within 6.5 Å of the reference amino residue’s Cα. The total number of native contacts is then the sum of the native contacts formed by all residues in the protein

To monitor and compare the unfolding process of the Cel7A proteins, MD simulations were also conducted at elevated temperature (475 K) for 15 ns, which was a sufficient length to observe initial unfolding events. From these simulations, we determined the total number of native contacts formed within the protein as a function of time and compared to the number of native contacts formed at 300 K (Fig. 14). As expected, the total number of native contacts formed in each cellulase was roughly constant at 300 K, i.e., not unfolding. *Tat*Cel7A exhibits a lower number of native contacts than either *Tre*Cel7A or *Tha*Cel7A (Fig. 14). When the temperature was elevated to 475 K, the number of contacts decreased at about the same rate for all three proteins as they unfolded, suggesting that they are equally sensitive to thermal unfolding.

Additional movie files show the initial unfolding of the protein structure during the 475 K simulations (see Additional files 2, 3 and, 4 for *Tat*Cel7A, *Tre*Cel7A, and *Tha*Cel7A, respectively). One region, residues 380–410 containing loops A4 and A2, was among the first parts of the protein to unfold, suggesting that this region may be an important ‘hotspot’ for initiation of protein unfolding. In loop A2, there is a short, surface exposed α-helix that maintained the helical structure longer in *Tha*Cel7A than in either *Tre*Cel7A or *Tat*Cel7A, suggesting higher regional thermal stability around loop A2 in *Tha*Cel7A. Another interesting observation is that the B3 loop in *Tre*Cel7A and *Tha*Cel7A (not *Tat*Cel7A) transiently flipped ~ 180° and adopted a conformation where the tip of the loop pointed towards subsite + 2, similar to the conformation observed in the structure of Cel7A from *Humicola grisea* var. *thermoidea* (PDB code 4CSI; [5]).

### Molecular evolution of Cel7A

**Fig. 15:**
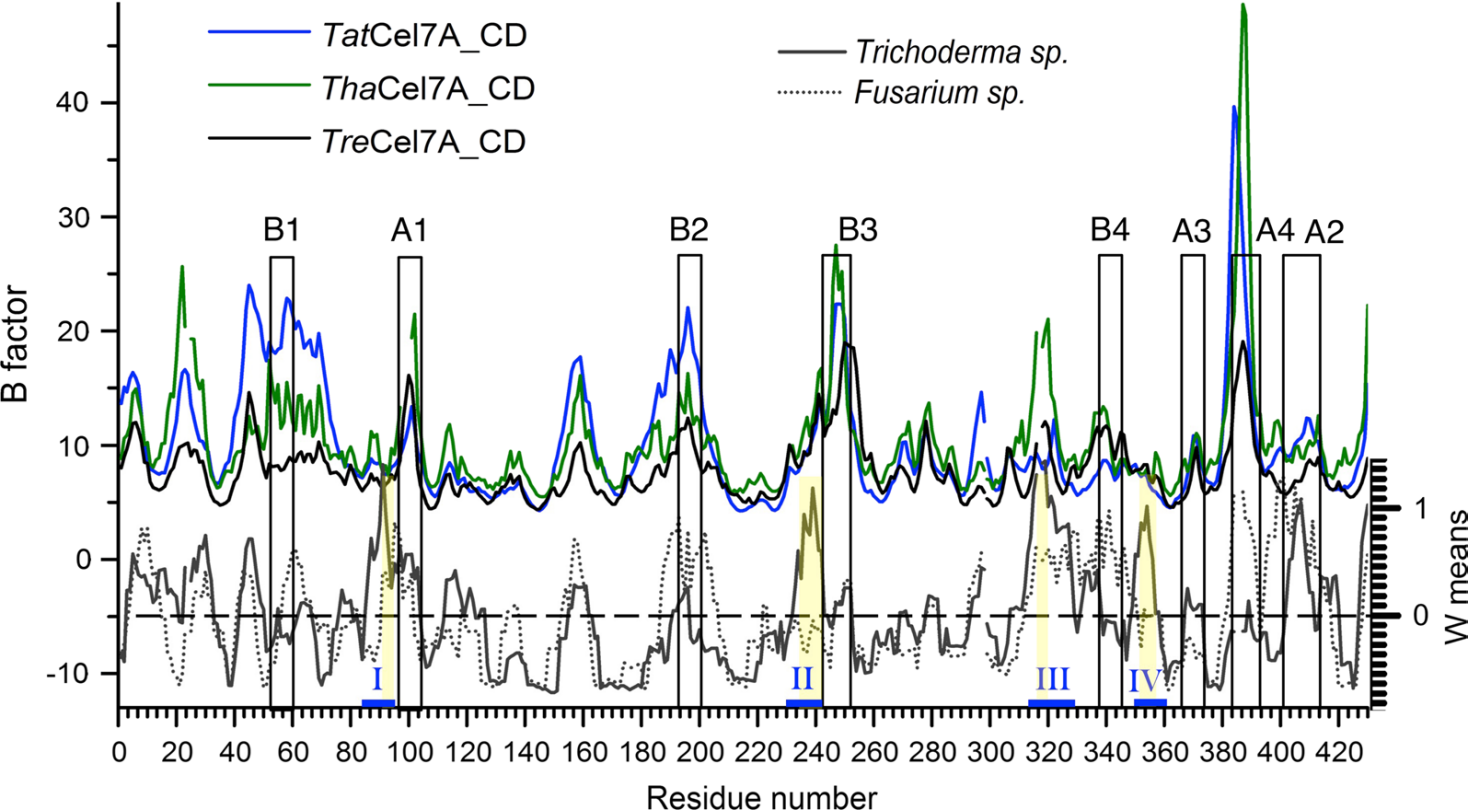
Temperature factors (B-factors) for Cel7A structures plotted over *W* mean scores from RCA analysis. The B-factors for amino acid Cα atoms of chain A in *Tre*Cel7A (4C4C), *Tha*Cel7A (2YOK), and *Tat*Cel7A (5O5D) structures are plotted against residue number in *Tat*Cel7A, aligned with the corresponding GH7 sequences (see Fig. 5). The scale for *W* mean scores from RCA analysis is on the right side of the graph. Sections of interest defined by RCA (reverse conservation analysis) are marked with blue lines and corresponding identifiers (I–IV), and residues with high *S* scores are marked in yellow

When comparing closely related orthologs, amino acid residues that modulate functional properties of an enzyme are expected to display higher diversity than other positions due to adaptation [35]. Therefore, distribution of amino acid variation was analyzed using RCA [35] of GH7 CBH sequences from two groups of related fungi within the order Hypocreales: *Trichoderma* spp. (11 sequences) versus *Fusarium* spp. and *Clonostachys rosea* (6 sequences) (Additional file 1: Figure S8). The orthologous status of the selected sequences was confirmed by a phylogenetic analysis (Additional file 1: Figure S9). We specifically analyzed the alignment for regions displaying signs of type 1 functional divergence (i.e., site conserved in one lineage but variable in the other [36]) in *Trichoderma* spp. Four sections were identified in the Cel7A alignment—I, II, III and IV (Fig. 12; (Additional file 1: Table S3)—that fulfilled the criteria (*W* mean score ≥ 1 in *Trichoderma* spp. and *W* mean score ≤ 1 in *Fusarium* spp.). All sites in sections I, II and III are located at the surface of the protein (Fig. 9). Section I is at a β-turn at one edge of the outer β-sheet, near the attachment of the linker. Section II comprises a short β-strand followed by a turn before loop B3 in the sequence and is located at the interface where the B2 and B3 loops are anchored. Section III includes the Gly317 insertion near the product-binding region mentioned above. Section IV includes an amphiphilic α-helix, where one side is buried; interestingly, three of the residue positions that display high amino acid diversity [*S* score ≥ 1, Met352, Ala355, Leu356 in *Tat*Cel7A, (Additional file 1: Table S3)] point into the hydrophobic core, just underneath the β-strand that carries the catalytic residues. In comparison with the Cel7A alignment of *C. rosea* and five *Fusarium* species, these sections (I, II, III, and IV) display signs of type 1 functional divergence, with the position conserved in *Fusarium* spp. but variable in *Trichoderma* spp. (Fig. 15) [36].

Figure 15 shows the *W* mean scores from RCA for *Trichoderma* spp. and *Fusarium* spp., plotted against residue number (in *Tat*Cel7A), compared with temperature factors (B-factors) for mainchain Cα atoms at corresponding positions in the crystal structures of *Tat*Cel7A (5O5D), *Tha*Cel7A (2YOK), and *Tre*Cel7A (4C4C). B-factors are indicators of protein flexibility and can be directly compared with RMSF from MD simulations. The three enzymes show almost the same pattern, with elevated B-factors in loop regions and the highest values in the A4 loop, all in good agreement with the MD simulation results (Fig. 11). The A4 loop is located at the exit of the tunnel and may affect release of the product, having contacts with section III (Fig. 9). Also noteworthy, loop A4 carries an N-glycosylation site near the product sites that is conserved in all three enzymes. Glycosylation at this site has been observed in structures of *Tre*Cel7A (Asn384) and *Tha*Cel7A (Asn380) but was not visible in the *Tat*Cel7A structures (Asn384).

## Discussion

Based on observations from initial hydrolysis of BMCC, synergistic conversion of pretreated biomass, and MD simulation, we suggest that the combination of the A1 and A3 loop motifs is the primary determinant for the observed differences in activity on cellulosic substrates. Initial hydrolysis of BMCC was most rapid with *Tat*Cel7A followed by *Tre*Cel7A and then *Tha*Cel7A, suggesting that the longer A1 loop, together with the weaker A3–B3 loop interaction (Tyr-Ala rather than Tyr–Tyr at the tip of loop A3), may be superior under these experimental conditions. Polikarpov et al. showed that deletion of three residues at the tip of the A1 loop in *Tha*Cel7A makes the entrance to the tunnel more open, and the replacement of one Tyr with Ala at the tip of loop A3 (relative to *Tre*Cel7A) increases the flexibility of the opposing B3 loop [21]. Our MD simulations of *Tre*Cel7A and *Tha*Cel7A, conducted here for comparison to *Tat*Cel7A, are in good agreement with those results. As with *Tha*Cel7A, the B3 loop of *Tat*Cel7A is more flexible and opens more frequently than in *Tre*Cel7A (Fig. 13). *Tha*Cel7A and *Tat*Cel7A share A3 loop features, whereas the A1 loop is similar in *Tat*Cel7A and *Tre*Cel7A. Thus, the most likely major determinant of the observed functional differences in initial crystalline cellulose hydrolysis is the longer A1 loop present in *Tat*Cel7A and *Tre*Cel7A.

Although *Tat*Cel7A showed higher *k*_cat_ in initial hydrolysis of BMCC, the processive model kinetic parameter fit to the progress curves did not reveal clear differences that could be readily correlated with protein structure and dynamics. The apparent processivity values (74–97) are in the same range, but somewhat higher than found by alternative methods (66–70) [17, 37, 38]. Also, both *k*_on_ and *k*_off_ values are higher in our case. However, the studies cited above employed other cellulose substrate preparations (e.g., reduced bacterial cellulose and Avicel) and may not be directly comparable. In our case, the *k*_off_ value is comparable or lower for *Tre*Cel7A_CD compared to the full-length enzyme. This is in contrast to Kont et al. who reported the opposite [38]. However, in a study by Cruys-Bagger et al. [32, 33], similar *k*_off_ values were found for *Tre*Cel7A and *Tre*Cel7A_CD. In that study, the authors suggested that the main energy barrier for dissociation is the release of the cellulose strand from the catalytic domain. In our current study, we find a slightly lower *k*_off_ value for the examined enzymes without a linker and CBM. We cannot explain this observation, but suspect that it may be related to the low DP of the substrate. With an estimated DP of around 120 glucose units and an apparent processivity of 70–90, the enzyme would, in most cases, ‘fall off’ when the entire cellulose chain has been hydrolyzed, rather than dissociating from the chain along the process.

When comparing the performance of the Cel7s in synergistic conversion of pretreated biomass to soluble sugar, both *Tat*Cel7A and *Tha*Cel7A gave higher yields of soluble sugar than *Tre*Cel7A, indicating that weaker A3–B3 interaction and, hence, higher B3 loop flexibility is beneficial to conversion. The length of the A1 loop may be of less importance, although *Tat*Cel7A, with the longer A1 loop, appears to be slightly more efficient than *Tha*Cel7A. Though our data implicates loops A1 and A3 in variable efficiency, we cannot rule out that other differences between the three enzymes may also influence their performance, such as linker length, glycosylation, or other residue substitutions that may affect the protein dynamics.

The B3 loop is anchored by disulfide bridges at both ends (Cys243 and Cys256 in *Tat*Cel7A) and is almost identical in sequence in the three enzymes, except for Asp-Asn conservative replacements at positions 249 and 250. In *Tat*Cel7A (and similarly in *Tha*Cel7A), Asn249 hydrogen bonds to the nearby Asp241, which would stabilize the loop and reduce fluctuations. Asn249 is replaced by Asp249 in *Tre*Cel7A, which, in some structures, forms a short distance, low-barrier hydrogen bond with Asp241 called an acid pair [39]. Such acid pair interactions are pH dependent and more distance restrained [40], which may contribute further to the restriction of B3 loop mobility in *Tre*Cel7A. Interestingly, the variable RCA defined section II before loop B3 includes the hydrogen-bonding partner, Asp241, and the nearby Ser239. The latter is replaced by Glu239 in *Tre*Cel7A, which is stabilized in turn by metal ion coordination together with His206. This may indicate that fine-tuning of B3 loop flexibility represents an important evolutionary target in *Trichoderma* spp. Cel7 proteins.

We were surprised to find that *Tat*Cel7A exhibits significantly lower activity against *p*NP-Lac, while it was about the same for *Tre*Cel7A and *Tha*Cel7A. The enzyme kinetics results show that this is mainly due to a significantly lower *k*_cat_ (Table 1). Also, the *K*_M_ value is slightly higher, giving a catalytic efficiency (*k*_cat_/*K*_M_) for *Tat*Cel7A of only about 25% compared to *Tre*Cel7A and *Tha*Cel7A. No obvious clues are evident from structural comparison, though, as to why that is the case. The three structures are practically identical at the subsites (− 2/− 1/+ 1) where *p*NP-Lac should bind for hydrolysis. However, *p*NP-Lac is an artificial chromogenic model substrate and may be a poor representative of function in Nature. Interestingly, a similar discrepancy in *p*NP-Lac activity has been reported previously for two close GH7 CBH orthologs from Amoebozoa [18]. Cel7A from *Dictyostelium discoideum* exhibited lower thermal stability and about half of the specific activity against *p*NP-Lac compared to *D. purpureum* Cel7A, despite 80% sequence identity.

The three enzymes showed similar pH dependence, with activity optimum around pH 4.5 and sensitivity to inactivation above neutral pH. This indicates that all the three species, *T. atroviride*, *T. reesei*, and *T. harzianum*, are adapted to biomass degradation at rather acidic conditions, without strong evolutionary pressure on their Cel7A enzymes towards action at higher pHs.

*Tat*Cel7A appears to be more temperature sensitive than either *Tre*Cel7A or *Tha*Cel7A, with a slightly lower temperature optimum and more rapid irreversible inactivation at elevated temperature. This is likely a function of fewer secondary structure interactions in *Tat*Cel7A relative to *Tre*Cel7A and *Tha*Cel7A, as observed by structural comparison and a lower number of native contacts found in the MD simulations. In particular, the A2–A4 region that appears to be a hotspot for initiation of unfolding seems to unfold faster in *Tat*Cel7A in the high-temperature MD simulations (see Additional file 2: Movie S1). Notably, though, the two regions on the backside of the protein where *Tat*Cel7A deviates structurally, i.e., near the linker attachment (13–17, 28–30) and around the Gly317 insertion (420–422), did not show any clear signs of unfolding more readily. Overall, the backside of the proteins remained remarkably stable throughout the high-temperature simulations, in contrast to large mobility of the extended loops along the active site.

The higher yield of soluble sugar obtained for *Tat*Cel7A vs. *Tre*Cel7A in the experiments on pretreated biomass suggests that this enzyme may be useful for industrial conversion of biomass. The lower temperature stability could be addressed by engineering a more stable variant inspired by *Tre*Cel7A or any other more thermostable GH7 CBH [5, 9]. The improvement of thermal stability of *Tre*Cel7A by directed evolution has recently been reported, where the most stable variant contains 18 mutations and exhibited a 10.4 °C increase in protein melting temperature [41]. Based on that study and the results herein, we propose that the primary region to target would be the A2–A4 region in order to stabilize the α-helix of the A2 loop while taking into account product – enzyme interactions at the exit of the tunnel. It should be noted, though, that irreversible inactivation depends not only on protein unfolding, but also on the exposure and aggregation of hydrophobic regions of the protein, which is difficult to predict.

## Conclusions

We have determined the three-dimensional structure and analyzed the properties of *Tat*Cel7A, the major secreted protein from *T. atroviride*, and compared these results to the close orthologs: *Tha*Cel7A and *Tre*Cel7A. All three proteins are very similar in sequence, structure, and several other aspects, yet, subtle differences are manifested in terms of stability, activity, and protein dynamics. Such differences, for example, in initial hydrolysis rates of BMCC and synergistic conversion of pretreated biomass, may lead to significant effects in the large-scale process applied for biomass conversion.

## Methods

### Preparation of *Trichoderma* Cel7 enzymes

The fungal strains *T. atroviride* IOC 4503 and *T. harzianum* IOC 3844 were obtained from the Culture Collection of Filamentous Fungi at the Oswaldo Cruz Institute (CCFF/IOC) in Brazil. They were grown on potato dextrose agar plates at 25 °C until dense sporulation developed (about 1 week) to produce fresh spores for culture inoculation. Submerged cultivation in distiller’s spent grain medium [42] with 1% w/v Avicel cellulose as a carbon source was undertaken for 6 days at 30 °C in a rotary incubator at 80 rpm; the cultivation took place in 2.8 L side-baffled Fernbach flasks (Bellco Glass Inc., Vineland, NJ, USA), each with 0.6 L medium containing: 6 g dry distillers spent grain, 9 g KH_2_PO_4_, 3 g (NH_4_)_2_PO_4_, 0.36 g MgSO_4_, and 0.36 g CaSO_4_. The pH was measured daily. On day 2, the pH dropped to around pH 3.5–3.8 for both fungi and was adjusted to pH 5 by addition of 2 g K_2_HPO_4_ to each flask. Upon harvest, the cultures were filtrated on Whatman GF/B glass fiber filters (~ 1 µm pore size) followed by 0.45 and 0.2 µm sterile filtration.

The culture filtrate was desalted on Bio-Gel P-6DG (BioRad; 500 mL column) to 10 mM potassium phosphate buffer, pH 6.0, then applied to a DEAE Sepharose Fast Flow column (GE Healthcare; CV = 200 mL) and eluted with a gradient up to 0.5 M NaCl in the same buffer. Fractions containing *p*NP-Lac activity were pooled, desalted, and applied to a SOURCE 30Q column (GE Healthcare; CV = 25 mL) eluted with a 10–500 mM potassium phosphate, pH 6.0, gradient. Fractions with activity against *p*NP-Lac were collected and subjected to SDS-PAGE analysis to estimate the purity of the Cel7 protein. The yield of purified enzyme per liter of culture was 70 mg for *Tat*Cel7A and 85 mg for *Tha*Cel7A.

*Tat*Cel7A_CD used for crystallization was prepared from the *T. atroviride* strain IMI 206040, kindly donated by Dr. Alexander Golubev (Petersburg Nuclear Physics Institute, Gatchina, Russia). Cultivation, protein purification, domain cleavage with papain and enzymatic N-deglycosylation were performed as previously described [3]. The solved crystal structure confirms that the protein sequence is identical to that of *Tat*Cel7A from *T. atroviride* strain IOC 4503, at least in the catalytic domain.

For all *Tre*Cel7A experiments except the PCS hydrolysis experiments, *Tre*Cel7A was obtained from *T. reesei* strain QM9414 as described [31, 43]. For the PCS hydrolysis experiments, *Tre*Cel7A was recombinantly produced in the *T. reesei* AST1116 constitutive expression system and purified to homogeneity as detailed in [44].

For preparation of the Cel7 catalytic domains, the CBM-linker portion of the full-length enzymes were removed by partial proteolysis using papain as previously described [3], followed by size-exclusion chromatography on a HiLoad Superdex 75 16/60 column (GE Healthcare) with 10 mM sodium acetate, pH 5.0, 0.15 M NaCl as eluent. Purified proteins were concentrated and stored in 10 mM sodium acetate, pH 5.0, at − 20 °C. Protein concentrations were determined spectrophotometrically at 280 nm using theoretical extinction coefficients calculated from amino acid sequences using the ProtParam web service (ExPASy ProtParam http://web.expasy.org/protparam/): *Tre*Cel7A, 86760 M^−1^ cm^−1^; *Tre*Cel7A_CD, 80550 M^−1^ cm^−1^; *Tat*Cel7A, 86760 M^−1^ cm^−1^; *Tat*Cel7A_CD, 80550 M^−1^ cm^−1^; *Tha*Cel7A, 90770 M^−1^ cm^−1^; *Tha*Cel7A_CD, 80550 M^−1^ cm^−1^.

### Temperature and pH dependence, enzyme kinetics and cellobiose inhibition

Hydrolytic activity measurements were carried out in triplicate in 96-well microtiter plates using *p*NP-Lac as substrate. Reaction mixtures of 150 µL contained 50 mM buffer (pH 3–7, phosphate-citrate; pH 7–8, potassium phosphate; pH 8–9, sodium borate), 2 mM of *p*NP-Lac and 0.15 µM of the enzyme (*Tre*Cel7A_CD, *Tha*Cel7A_CD, or *Tat*Cel7A_CD). The reaction was quenched by adding 150 µL of 0.5 M sodium carbonate, followed by measurement of absorbance at 405 nm using an Eon Multiplate Reader. The rate of *p*NP release was calculated using an extinction coefficient of 18.3 mM^−1^ cm^−1^.

The pH dependence of hydrolytic activity was determined in the range of pH 3.0–8.0. The reactions were incubated at 30 °C for 30 min. In pH stability experiments, the enzymes were pre-incubated at 40 °C at pHs from pH 3.0–9.5 for 20 h, followed by *p*NP-Lac activity measurement at 30 °C and pH 4.5 using a 30-min incubation.

For temperature dependence of activity, the reactions were incubated in the temperature range of 20–75 °C for 1 h at pH 4.5. The reaction components were pre-cooled and mixed on ice, then transferred into the thermostat equilibrated at the desired temperature. For assessment of thermal inactivation, the enzymes were pre-incubated at 60, 65, and 70 °C at pH 4.5. Aliquots were taken at indicated time points up to 90 min and cooled on ice, followed by determination of residual hydrolytic activity against *p*NP-Lac at 30 °C, pH 4.5, and 1 h incubation time.

Experiments for determination of enzyme kinetics parameters *V*_max_ and *K*_M_ for *p*NP-Lac as substrate and inhibition constants *K*_i_ for cellobiose were done in 96-well microtiter plates as described above. Reaction mixtures containing *Tre*Cel7A_CD, *Tha*Cel7A_CD, or *Tat*Cel7A_CD (0.12, 0.22, 0.12 µM, respectively), 50 mM sodium phosphate citrate buffer, pH 4.5, and *p*NP-Lac at 0.1, 0.2, 0.4, 0.67, 1.2, 2, 3, 4, 5, and 6.7 mM concentration, without and with 100 µM cellobiose, were incubated for 1 h at 30 °C. Nonlinear regression fitting was accomplished using the Excel Solver add-in (Microsoft, Richmond, WA, USA). Weighted squared residuals were calculated for each data point using a statistical weighting scheme, [(*v*_obs_ − *v*_calc_)^2^/*v*_calc_], where *v*_obs_ is the observed reaction rate, and *v*_calc_ is the rate calculated from kinetic parameters (*V*_max_, *K*_M_, *K*_i_). The kinetic parameters were fit towards the minimized sum of residuals using the GRG nonlinear solving method within Solver. Mixed inhibition was first evaluated. In all cases, the uncompetitive *K*_i_ was more than an order of magnitude higher than the competitive *K*_i_, indicating that cellobiose acted as a competitive inhibitor. Therefore, the final values shown in Table 1 were derived by fitting the data to the Michaelis–Menten expression for competitive inhibition (see Additional file 1: Figure S2). The RMSD between *v*_calc_ and *v*_obs_ was used as indicator of experimental error (3.4, 4.0 and 2.4% for *Tre*Cel7A_CD, *Tat*Cel7A_CD, and *Tha*Cel7A_CD, respectively).

### Initial hydrolysis of cellulose

The initial hydrolysis of cellulose was measured using Biosensor equipment at Roskilde University, Denmark. Bacterial microcrystalline cellulose (BMCC) from *Acetobacter xylinum* was prepared from bacterial cellulose (BC) extracted from commercially available Nata de Coco as described [45]. The degree of polymerization of such BMCC has been determined at 114 glucose units [45]. Hydrolysis of BMCC was monitored by cellobiose product formation. The concentration of cellobiose was measured in real time with cellobiose dehydrogenase-modified carbon paste electrodes as described in detail by Cruys-Bagger et al. [32, 46]. The sensor had a response time and lower detection limit of 4 s and 60 nM, respectively. All reactions were carried out in 50 mM sodium acetate pH 5.0 at 25 °C with stirring. The reaction mixture contained 3.3 g/L of BMCC and 50 nM enzyme (*Tre*Cel7A, *Tre*Cel7A_CD, *Tha*Cel7A, *Tat*Cel7A, and *Tat*Cel7A_CD). The experimental data (time interval 0–200 s) was fit to the processive model shown in Additional file 1: Figure S4A. The model consists of three rate-constants, *k*_on_, *k*_cat_, and *k*_off_, and an apparent processivity parameter, *n*. For further detail, see Additional file 1.

### Pretreated corn stover (PCS) hydrolysis

Corn stover was harvested in 2009 in Hurley County, SD, USA, and was knife milled to pass a 19 mm (0.75 in) round screen and stored indoors in 200 kg lots at NREL (National Renewable Energy Laboratory, Golden, CO, USA). The compositional analysis of the native corn stover is given by Chen et al. [47]. Dilute acid pretreated corn stover (PCS) was prepared and analyzed by NREL standard laboratory analytical procedures [48], with PCS composed of 64.2% dry weight glucan. The PCS substrate was suspended in 20 mM sodium acetate buffer at pH 5.0. Digestions were conducted at 40 °C in high-performance liquid chromatography (HPLC) vials placed in a rotator at 10 rpm up to 96 h. An amount of PCS substrate equivalent to 8.5 mg of glucan was added to the enzymatic cocktail consisting of each of the GH7 CBHs, endoglucanase I from *T. longibrachiatum* (Megazyme Co., Bray, Ireland), and β-glucosidase from *Aspergillus niger* (Megazyme Co., Bray, Ireland) at a concentration of 28, 1.9, and 0.5 mg protein/g of glucan, respectively. The ratio and dosage of enzymes used here represent one of the standard conditions developed and used at NREL to assay the performance of Cel7 enzymes in NREL PCS conversion [49, 50]. Adjustment of the biomass assay aliquots to 1.7 mL final volume resulted in a cellulose concentration of 5.0 mg/mL and a GH7 CBH concentration of 0.14 mg/mL, corresponding to 2.5 µM for *Tre*Cel7A. Sugar analyses were performed by HPLC as reported in [44]. Experiments were performed in duplicate.

### X-ray crystallography

Crystallization experiments were carried out with the deglycosylated catalytic domain *Tat*Cel7A_CD. Screening for crystallization conditions was performed in 96-well sitting drop trays using a Mosquito crystallization robot (TTP Labtech, UK). The most promising crystallization hits were obtained at room temperature with Hampton polyethylene glycol (PEG)/Ion screen. The final optimized conditions contained 5 mM NiCl_2_, 0.1 M HEPES pH 7.0, and 20% w/v PEG 3350 as a precipitant. Crystals used for data collection were grown by sitting drop vapor diffusion under the same conditions after 1:1 mixing of precipitant with 4.8 mg/mL *Tat*Cel7A_CD in 20 mM Bis–Tris buffer, pH 7.0. Cellobiose was added to the crystallization drops for the APO structure but is not seen in the structure. The SG3 structure complex was obtained from co-crystallization drops with 5 mM 4,4′-dithio-cellotriose.

X-ray diffraction data were collected at 100 K at the synchrotron beamline ID23-1, ESRF, Grenoble, France, as indicated in Table 3. The data were integrated with XDS [51] and scaled using the programs Scala and Aimless in the CCP4 suite [52]. The initial *Tat*Cel7A_CD structure model was solved by molecular replacement using PHASER [53] and a structure of *Tre*Cel7A_CD as the search model (PDB code 1CEL).

REFMAC5 [54] was used for structure model refinements, and manual model rebuilding was performed with Coot [55, 56] using maximum likelihood sigma-average-weighted 2*F*_o_–*F*_c_ electron density maps [56]. For cross-validation by *R* and *R*_free_ calculations, 5% of the data were excluded from the structure refinement [57]. Solvent molecules were automatically added using the automatic water picking function in the ARP/wARP package [58]. Picked water molecules were selected or discarded manually by visual inspection of 2*F*_o_–*F*_c_ and *F*_o_–*F*_c_ electron density maps. The coordinates for the two final *Tat*Cel7A_CD structure models and the structure factors have been deposited in the Protein Data Bank (http://wwpdb.org/) with accession codes 5O5D and 5O59.

### Molecular dynamics simulations

For the catalytic domain of each enzyme (*Tat*Cel7A, *Tre*Cel7A, and *Tha*Cel7A), three ligand-bound states were modeled: without a ligand (no ligand), bound to cellononaose (ligand), and bound to a cellulose Iβ microfibril (microfibril) (Fig. 10). The cellulase structures used for MD simulations were obtained from crystal structures deposited in the Protein Data Bank: PDB ID 4C4C for *Tre*Cel7A [59], 2YOK for *Tha*Cel7A [21], and 5O5D for *Tat*Cel7A. The three simulations of *Tre*Cel7A at 300 K have been previously reported [3] and are presented here again for direct comparison to *Tha*Cel7A and *Tat*Cel7A dynamics. Additionally, we carried out a set of MD simulations at an elevated temperature, 475 K, considering each cellulase in the ligand-free “Apo” state in solution, to examine the unfolding process of the enzymes and to locate regions vulnerable to increased temperature (hotspots).

To build the *Tre*Cel7A apo simulation, the cellononaose ligand was removed from the active site of the catalytic domain. For the cellononaose-bound state, the cellononaose ligand from 4C4C was retained from the crystal structure (4C4C), occupying the active site from − 7 to + 2 sites (Fig. 10). The *Tre*Cel7A microfibril complex was constructed by docking the cellononaose-bound catalytic domain on the hydrophobic face of the cellulose 1β crystal matrix, where a single chain had been decrystallized as previously described [3]. In each *Tre*Cel7A case, the mutated Gln217 was reverted to the wild-type glutamic acid. Additional details of the modeling procedure for the *Tre*Cel7A simulations can be found in our previous work [3]. The *Tha*Cel7A and *Tat*Cel7A ligand-free simulation sets were constructed from the apo crystal structures. The cellononaose-bound *Tha*Cel7A and *Tat*Cel7A models were constructed by aligning the protein backbone with *Tre*Cel7A (4C4C) and adopting the coordinates of the 4C4C cellononaose; structural alignment was performed using PyMOL [60]. The *Tha*Cel7A and *Tat*Cel7A microfibril complexes were constructed as described for *Tre*Cel7A above and previously [3].

In each model, only the catalytic domains of cellulases were simulated, excluding the glycosylated linker and the carbohydrate-binding module. Additionally, the glycans attached to the catalytic domains were omitted from the models, as they have relatively limited effects on the protein dynamics over MD-simulation time scales [61]. pKa calculations, using the H++ webserver, and visual inspection were used to determine the protonation states of the titratable residues at pH 5.0 with internal and external dielectrics of 10 and 80, respectively [62–64]. Disulfide bonds were defined according the PDB structures. CHARMM was used to construct and explicitly solvate the systems with the water molecules (80 Å × 80 Å × 80 Å for no ligand and ligand systems; 135 Å × 100 Å × 90 Å for the microfibril complexes) [65]. Na^+^ ions were added to ensure the charge neutrality of the system, avoiding the self-energy artifact [66, 67].

Minimization and equilibration simulations were conducted in CHARMM using the CHARMM36 force field to define the protein and carbohydrate behavior and the modified TIP3P force field for water [68–73]. Minimization of each system was conducted in three steps: (1) keeping the protein, the ligand (if present), and the microfibril (if present) fixed and allowing the water molecules to move freely, then (2) keeping only the protein fixed, allowing the remainder of the system to move freely, and (3) allowing every atom in the system to move freely without any restraint. Each of the three minimization steps used 1000 steps of steepest decent (SD) minimization. Following minimization, the systems were heated from 100 to 300 K in the *NVE* ensemble for 20 ps using 50 K temperature increments every 4 ps. The systems were then density equilibrated in the *NPT* ensemble at 300 K for 100 ps. Data collection simulations of 100 ns were conducted using NAMD in the *NVT* ensemble at 300 K with a time step of 2 fs [65, 74]. Evaluation of the RMSD of the protein backbones, compared to their positions following density equilibration, indicates 100 ns is sufficient to reach a local equilibrium (Additional file 1: Figure S10). Long-range electrostatic calculations used a non-bonded cutoff distance of 10 Å, a switching distance of 9 Å, and a non-bonded pair list distance of 12 Å. The SHAKE algorithm was used to fix the hydrogen distances during all simulations. For microfibril complexes, during heating, density equilibration and production simulation, the bottom layer of the cellulose crystal was harmonically restrained with a force constant of 1 kcal/mol/Å^2^ to prevent twisting of the microfibril, which occurs when the degree of polymerization is low.

To initiate the high temperature simulations, we first conducted three independent 50-ns MD simulations of each apo enzyme (9 total simulations) at 300 K in the *NVT* ensemble using NAMD. The high temperature simulations were started from 10 ns, 300 K equilibrated snapshots of each enzyme. High-temperature simulations were conducted in NAMD at 475 K for 15 ns each; all other simulation parameters were as described above. Again, three independent simulations of each enzyme were performed to obtain statistically meaningful structural insight. VMD was used to visualize the trajectories of the high temperature simulations and define the thermally unstable regions of the enzymes. The native contact analysis described above was conducted in CHARMM using the COORdinate DMAT (distance matrix) command.

### Phylogenetic analysis

GH7 protein sequences were retrieved by pBLAST search with the *Tre*Cel7A full-length sequence (UniProtKB-P62694) in NCBI and individual species genome databases. Available sequences of both CBHs and EGs from *Trichoderma* spp., *Fusarium* spp. and *C. rosea* were selected, and one sequence from *Acremonium strictum* was included as an outgroup, resulting in a set of 28 GH7 orthologs. The amino acid sequences were aligned by ClustalW using MEGA7 software [75], and regions flanking the GH7 domain were trimmed off (signal peptide, before Gln 1 of *Tat*Cel7A; linker-CBM, after Thr429 of *Tat*Cel7A). The evolutionary history was inferred using the minimum evolution method [76] and bootstrap phylogeny testing with 2000 replicates. The evolutionary distances were computed using the Dayhoff matrix based method [77] and are in the units of the number of amino acid substitutions per site. The minimum evolution tree was searched using the close-neighbor-interchange (CNI) algorithm [78] at a search level of 1. The neighbor-joining algorithm [79] was used to generate the initial tree. All positions containing gaps and missing data were eliminated. There were a total of 349 positions in the final dataset.

### Reverse conservation analysis (RCA)

A subset of 17 GH7 CBH protein sequences, including 11 sequences from *Trichoderma* spp. and six sequences from *Fusarium* spp. and *C. rosea*, was selected. The GH7 CBH catalytic domains were realigned by ClustalW using MEGA7 software [75], followed by indel elimination. This alignment was analyzed by RCA as described earlier [35]. In short, Rate4Site (Version 2.01) was used to calculate the degree of conservation (*S* score) for each amino acid position using the empirical Bayesian method [80, 81]. A sliding window-average (*n* = 7) *S* score was plotted (*W* mean score) and significant peaks were defined by intensity (*I*) values of 1 [35].

## Additional files

**Additional file 1: Figure S1.** SDS-PAGE analyses of *T. atroviride* culture filtrate and purified *Trichoderma* spp. Cel7A enzymes. **Figure S2.** Substrate dependence plots and Hanes-Wolff plots from enzyme kinetics experiments with *Tat*Cel7A, *Tha*Cel7A and *Tre*Cel7A, using *p*NP-Lac as substrate and cellobiose as inhibitor. Additional information regarding the mathematical model for quasi-steady state kinetics of processive cellulose hydrolysis by GH7 cellobiohydrolases and the derivation of kinetic parameters by non-linear regression fitting to real-time progress curves of the initial stage of cellulose hydrolysis. **Figure S3.** A) Real-time progress curves. B) Derivative of the progress curves in A). **Figure S4.** A) Simplified reaction scheme for a processive cellulase. B) Illustration of the molecular steps involved in the reaction scheme. **Figure S5.** Non-linear regression fit to real-time progress curves. **Figure S6.** Bar diagram of kinetic parameters derived from initial hydrolysis of BMCC. Additional information regarding correlation of kinetic parameters derived by non-linear regression fit to initial hydrolysis data. **Table S1.** Parameter correlation matrix for *Tre*Cel7A. **Figure S7.** Kinetic parameter fit to simulated data with 2.5% random noise added, and to experimental data recorded for *Tre*Cel7A during initial hydrolysis of BMCC. **Table S2.** Comparison of kinetic parameters from the fit to simulated data with 2.5% random noise, and to experimental data recorded for *Tre*Cel7A during initial hydrolysis of BMCC. **Figure S8.** Sequence alignment of the GH7 CBH catalytic domains used for RCA analysis. **Figure S9.** Phylogenetic tree of GH7 catalytic domain protein sequences from *Trichoderma* spp. and *Fusarium* spp. **Table S3.** S scores from RCA analysis for residues of interest for *Tat*Cel7A, *Tha*Cel7A and *Tre*Cel7A. Additional MD simulation results **Figure S10.** RMSD as a function of time for each 100-ns, ligand-bound MD simulation of *Tat*Cel7A, *Tha*Cel7A and *Tre*Cel7A catalytic domains.

**Additional file 2: Movie S1.** Movie of *Tat*Cel7A_CD initial protein unfolding during 15-ns MD simulations at high temperature (475 K). The movie shows three individual MD runs side-by-side for the same protein, in two views. The top row shows the “front” of the enzyme, and the bottom row shows the “backside”.

**Additional file 3: Movie S2.** Movie of *Tre*Cel7A_CD initial protein unfolding during 15-ns MD simulations at high temperature (475 K). The movie shows three individual MD runs side-by-side for the same protein, in two views. The top row shows the “front” of the enzyme, and the bottom row shows the “backside”.

**Additional file 4: Movie S3.** Movie of *Tha*Cel7A_CD initial protein unfolding during 15-ns MD simulations at high temperature (475 K). The movie shows three individual MD runs side-by-side for the same protein, in two views. The top row shows the “front” of the enzyme, and the bottom row shows the “backside”.

## Abbreviations

BCA: biocontrol agent
BMCC: bacterial microcrystalline cellulose
CBH: cellobiohydrolase
CBM: carbohydrate binding module
CD: catalytic domain
EG: *endo*-1,4-β-d-glucanase
DP: degree of polymerization
GH6: glycoside hydrolase family 6
GH7: glycoside hydrolase family 7
MD: molecular dynamics
PCS: pretreated corn stover
*p*NP-Lac: *p*-nitrophenyl β-lactoside
RCA: reverse conservation analysis
RMSD: root mean square deviation
RMSF: root mean square fluctuation
*Tat*Cel7A: *Trichoderma atroviride* Cel7A
*Tha*Cel7A: *Trichoderma harzianum* Cel7A
*Tre*Cel7A: *Trichoderma reesei* Cel7A
