# Supplementary material for: Correlation of structure, function and protein dynamics in GH7 cellobiohydrolases from Trichoderma atroviride, T. reesei and T. harzianum
Source: Biotechnol Biofuels. 2018 Jan 13;11:5. doi: 10.1186/s13068-017-1006-7 (PMC5766984; doi:10.1186/s13068-017-1006-7)
Supplement: Supplementary file 1 — Additional file 1: Figure S1. SDS-PAGE analyses of T. atroviride culture filtrate and purified Trichoderma spp. Cel7A enzymes. Figure S2. Substrate dependence plots and Hanes-Wolff plots from enzyme kinetics experiments with TatCel7A, ThaCel7A and TreCel7A, using pNP-Lac as substrate and cellobiose as inhibitor. Additional information regarding the mathematical model for quasi-steady state kinetics of processive cellulose hydrolysis by GH7 cellobiohydrolases and the derivation of kinetic parameters by non-linear regression fitting to real-time progress curves of the initial stage of cellulose hydrolysis. Figure S3. A) Real-time progress curves. B) Derivative of the progress curves in A). Figure S4. A) Simplified reaction scheme for a processive cellulase. B) Illustration of the molecular steps involved in the reaction scheme. Figure S5. Non-linear regression fit to real-time progress curves. Figure S6. Bar diagram of kinetic parameters derived from initial hydrolysis of BMCC. Additional information regarding correlation of kinetic parameters derived by non-linear regression fit to initial hydrolysis data. Table S1. Parameter correlation matrix for TreCel7A. Figure S7. Kinetic parameter fit to simulated data with 2.5% random noise added, and to experimental data recorded for TreCel7A during initial hydrolysis of BMCC. Table S2. Comparison of kinetic parameters from the fit to simulated data with 2.5% random noise, and to experimental data recorded for TreCel7A during initial hydrolysis of BMCC. Figure S8. Sequence alignment of the GH7 CBH catalytic domains used for RCA analysis. Figure S9. Phylogenetic tree of GH7 catalytic domain protein sequences from Trichoderma spp. and Fusarium spp. Table S3. S scores from RCA analysis for residues of interest for TatCel7A, ThaCel7A and TreCel7A. Additional MD simulation results Figure S10. RMSD as a function of time for each 100-ns, ligand-bound MD simulation of TatCel7A, ThaCel7A and TreCel7A catalytic domains. [file 13068_2017_1006_MOESM1_ESM.pdf]

Additional file 1

Correlation of structure, function and protein dynamics in GH7 cellobiohydrolases from *Trichoderma atroviride, T. reesei* and *T. harzianum*

Anna S. Borisova[1,2], Elena V. Eneyskaya[2], Suvamay Jana[3], Silke F. Badino[4], Jeppe Kari[4], Antonella Amore[5], Magnus Karlsson[6], Henrik Hansson[1], Mats Sandgren[1], Michael E. Himmel[5], Peter Westh[4], Christina M. Payne[3,*,§], Anna A. Kulminskaya[2,7,*], Jerry Ståhlberg[1,*]

[1] Swedish University of Agricultural Sciences, Department of Molecular Sciences, P.O. Box 7015, SE-750 07 Uppsala, Sweden.
[2] B.P. Konstantinov Petersburg Nuclear Physics Institute, National Research Centre «Kurchatov Institute», Orlova roscha, Gatchina, Leningrad region, 188300, Russia.
[3] University of Kentucky, Department of Chemical and Materials Engineering, 177 F. Paul Anderson Tower, Lexington, KY 40506-0046, USA.
[4] Roskilde University, Department of Science and Environment, 1 Universitetsvej, DK-4000 Roskilde, Denmark.
[5] National Renewable Energy Laboratory, Biosciences Center, 15013 Denver West Parkway, Golden, CO 80401, USA.
[6] Swedish University of Agricultural Sciences, Department of Forest Mycology and Plant Pathology, P.O. Box 7026, SE-750 07 Uppsala, Sweden.
[7] Peter the Great St. Petersburg Polytechnic University, Department of Medical Physics, St Petersburg, Russia.
[§] Current address (CMP): Division of Chemical, Bioengineering, Environmental, and Transport Systems, National Science Foundation, Alexandria, VA, USA

* Corresponding authors: Jerry Ståhlberg <jerry.stahlberg@slu.se>, Christina Payne <christy.payne@uky.edu>, Anna Kulminskaya <kulminskaya_aa@pnpi.nrcki.ru>

In addition to this document, movies are provided in three separate files [Additional file 2, 3, 4], which show the initial protein unfolding during 15-ns MD simulations at high temperature (475 K) for Cel7A from *Trichoderma atroviride, T. reesei* and *T. harzianum*, respectively. Each movie shows three indivudal MD runs side-by-side for the same protein, in two views. The top row shows the "front" of the enzyme, and the bottom row shows the backside.

This Additional file 1 contains:

Figure S1. SDS-PAGE analyses of *T. atroviride* culture filtrate and purified *Trichoderma* spp. Cel7A enzymes.

Figure S2. Substrate dependence plots and Hanes-Wolff plots from enzyme kinetics experiments with *Tat*Cel7A, *Tha*Cel7A and *Tre*Cel7A, using pNP-Las as substrate and cellobiose as inhibitor.

Additional information regarding the mathematical model for quasi-steady state kinetics of processive cellulose hydrolysis by GH7 cellobiohydrolases and the derivation of kinetic parameters by non-linear regression fitting to real-time progress curves of the initial stage of cellulose hydrolysis.

Figure S3. A) Real-time progress curves. B) Derivative of the progress curves in A).

Figure S4. A) Simplified reaction scheme for a processive cellulase. B) Illustration of the molecular steps involved in the reaction scheme.

Figure S5. Non-linear regression fit to real-time progress curves.

Figure S6. Bar diagram of kinetic parameters derived from initial hydrolysis of BMCC.

Additional information regarding correlation of kinetic parameters derived by non-linear regression fit to initial hydrolysis data.

Table S1. Parameter correlation matrix for *Tre*Cel7A.

Figure S7. Kinetic parameter fit to simulated data with 2.5 % random noise added, and to experimental data recorded for *Tre*Cel7A during initial hydrolysis of BMCC.

Table S2. Comparison of kinetic parameters from the fit to simulated data with 2.5 % random noise, and to experimental data recorded for *Tre*Cel7A during initial hydrolysis of BMCC.

Figure S8. Sequence alignment of the GH7 CBH catalytic domains used for RCA analysis.

Figure S9. Phylogenetic tree of GH7 catalytic domain protein sequences from *Trichoderma* spp. and *Fusarium* spp.

Table S3. S scores from RCA analysis for residues of interest for *Tat*Cel7A, *Tha*Cel7A and *Tre*Cel7A.

Additional MD simulation results

Figure S10. RMSD as a function of time for each 100-ns, ligand-bound MD simulation of *Tat*Cel7A, *Tha*Cel7A and *Tre*Cel7A catalytic domains.

SDS-PAGE analysis of *T. atroviride* culture filtrate and purified enzymes

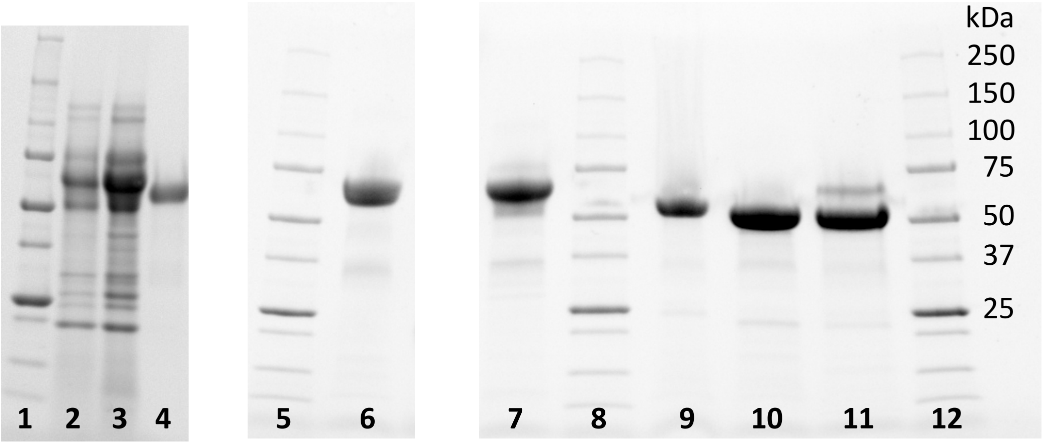

Figure S1. SDS-PAGE analyses. Lanes 1, 5, 8 and 12: Molecular weight marker proteins; approximate mass in kDa is indicated to the right. Lane 2: Culture filtrate from T. atroviride strain IMI 206040 grown for 6 days in distiller's spent grain medium with 1% Avicel cellulose as carbon source. Lane 3: Culture filtrate from T. atroviride strain IOC 4503 grown for 6 days. Lane 4: Purified T. atroviride Cel7A catalytic domain (TatCel7A_CD) after papain cleavage. Lane 6: Purified T. reesei Cel7A full-length (TreCel7A). Lane 7: Purified TatCel7A full-length. Lane 9: Purified TreCel7A_CD. Lane 10: Purified T. harzianum Cel7A catalytic domain (ThaCel7A_CD). Lane 11: Purified TatCel7A_CD.

Enzyme kinetics and cellobiose inhibition

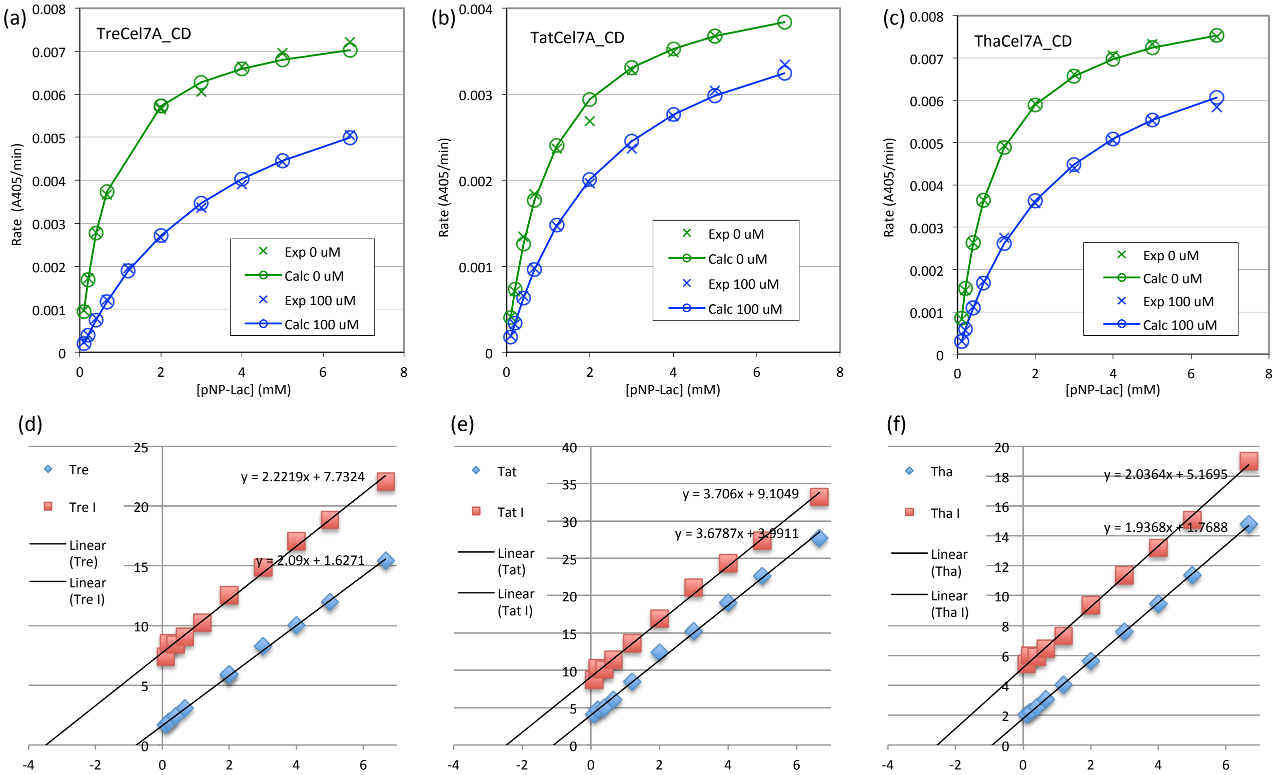

Figure S2. Enzyme kinetics with *p*NP-Lac as substrate, without and with 100 uM cellobiose, at 30°C, pH 4.5.
Top: Substrate dependence plots for *Tre*Cel7A_CD (a), *Tat*CelA_CD (b), and *Tha*Cel7A_CD (c).
Bottom: Hanes-Wolff plots ([S]/v = f [S]) for *Tre*Cel7A_CD (d), *Tat*CelA_CD (e), and *Tha*Cel7A_CD (f).

Modeling of GH7 cellobiohydrolase cellulase kinetics

Experimental conditions:

- Substrate: 3.3 g/L BMCC
- Enzyme: 50 nM

Measurement:

- Real-time measurement of cellobiose with amperometric enzyme biosensor. Biosensors where based on *Phanerochaete chrysosporium* cellobiose dehydrogenase (PcCDH)

Criteria for quasi-steady state – Choosing the time-interval

Selection of the experimental timescale is particularly important in cellulase kinetics, as both short- and long-term effects contribute to a drop in the reaction rate with time (1-4). Hence, choosing a time interval for the regression analysis will affect the derived rate constants. To choose a time-scale, we plotted the derivative of the real-time biosensor measurements (see Figure S3B). As shown in Figure S3B, a quasi-steady-state regime is established after approximately 200 seconds. We will, in the following, use this interval (0-200 s) for our non-linear regression analysis.

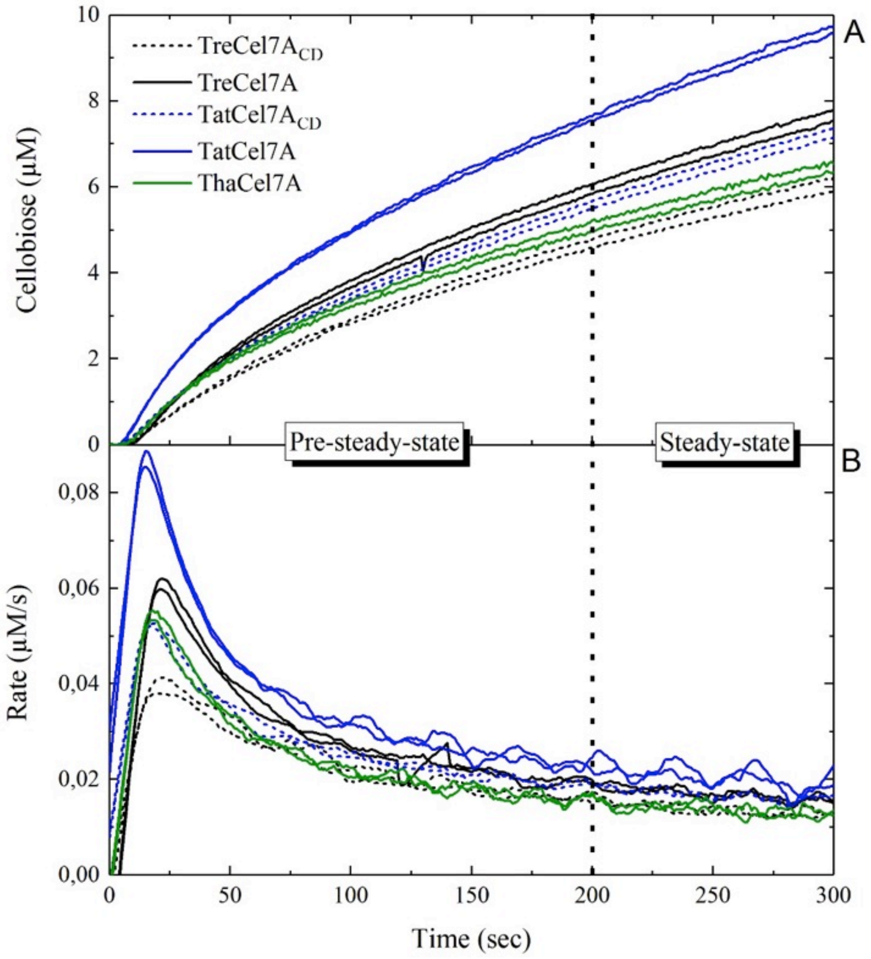

Figure S3. A) Real-time progress curves of *Tre*Cel7A, *Tat*Cel7A, *Tha*Cel7A and the catalytic domain of *Tre*Cel7A (*Tre*Cel7A$_{CD}$) and *Tat*Cel7A (*Tat*Cel7A$_{CD}$). B) Derivative of the data in A. The enzyme concentration was 50 nM, and the BMCC load was 3.3 g/L.

Non-linear regression analysis of biosensor measurement

The experimental data was fit to the processive model scheme shown in Figure S4A. The model consists of three rate-constants, k_{on}, k_{cat}, and k_{off}, and an apparent processivity parameter, n. For further detail see Praestgaard, *et al.* (5).

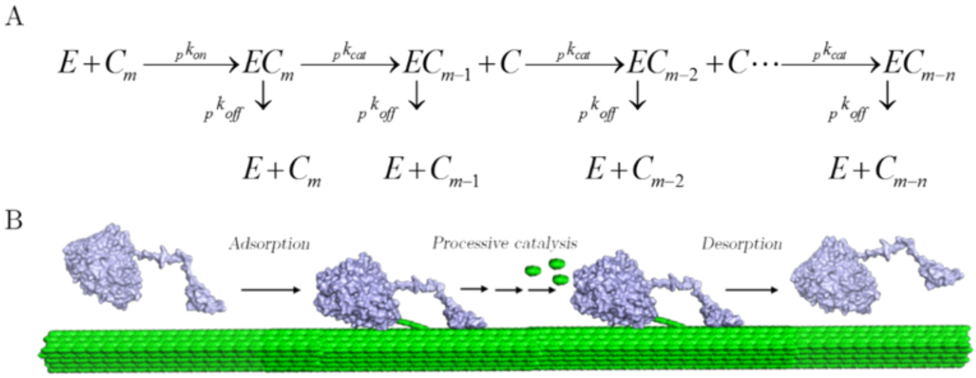

Figure S4. Simplified reaction scheme for a processive cellulase (A) and an illustration of the molecular steps involved in this scheme (B). Reaction scheme (A) is taken from Praestgaard, et al. (5).

All experiments were done in duplicate using two different biosensors, which we will call biosensor A and B. Individual fits of the experimental data obtained with biosensor A and B and the average parameters are given in Table 1 (in the main text of the article) together with the standard deviation between these independent derived parameters.

Curve fit (fit up to 200 seconds)

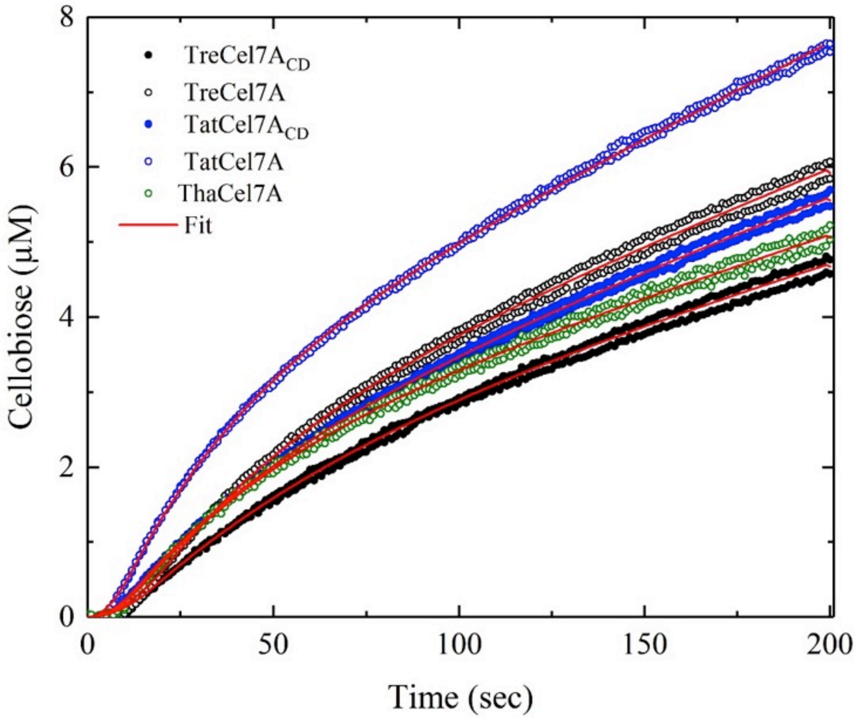

Figure S5. Nonlinear regression of data for *Tre*Cel7A, *Tat*Cel7A, *Tha*Cel7A and the catalytic domains of *Tre*Cel7A (*Tre*Cel7A$_{CD}$) and *Tat*Cel7A (*Tat*Cel7A$_{CD}$). The enzyme concentration was 50 nM and the substrate load was 3.3 g/L BMCC. Circles represent experimental data points from two measurments for each enzyme, and the respective fit is shown as a red line..

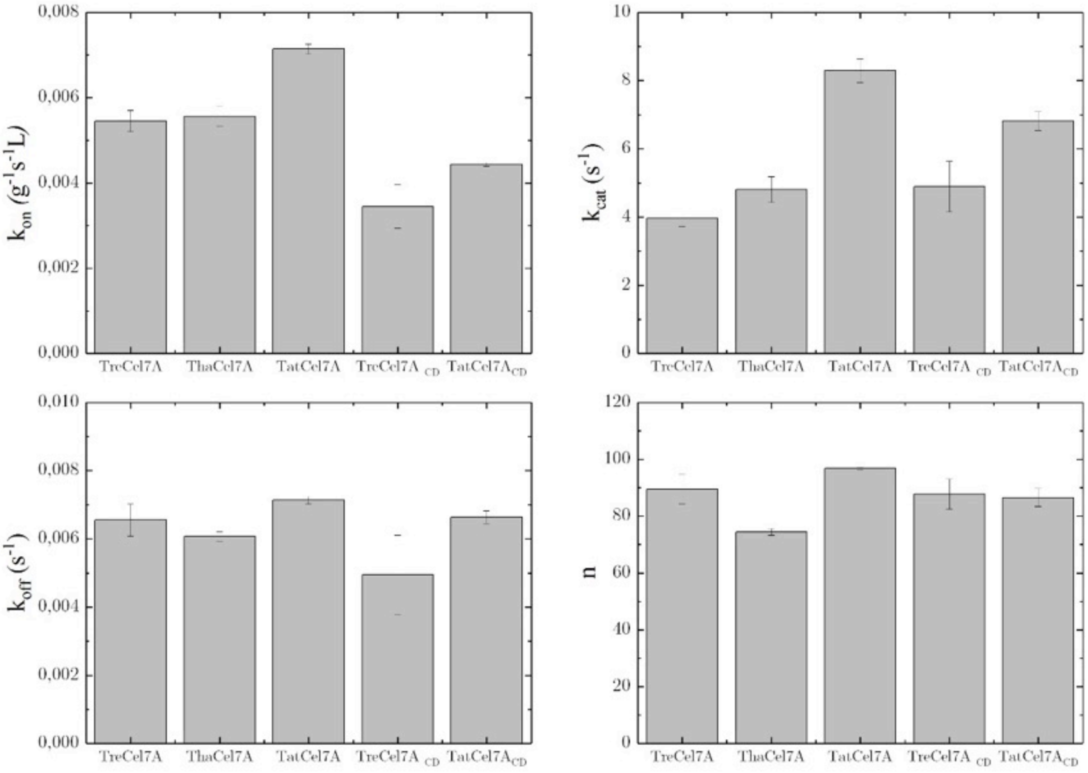

Figure S6. Kinetic parameters derived from initial hydrolysis of BMCC (see Figure S4).

Parameter correlation test

To test the parameter correlation a preliminary analysis was conducted to calculate the correlation matrix for the parameters derived for *Tre*Cel7A (see Table S1).

Table S1. Correlation matrix for *Tre*Cel7A

	k_{on}	k_{cat}	k_{off}	n
k_{on}	1	-0.96	-0.73	-0.97
k_{cat}		1	-0.63	-0.88
k_{off}			1	-0.87
n				1

The correlation matrix showed significant negative correlation between k_{on} and k_{cat} as well as k_{on} and n. Hence, a lower k_{on} value could to some degree be compensated by higher k_{cat} or n and vice versa. We note that the non-linear regression analysis, was done individually for two independent progress curves and the best-fit parameters from the individual fit gave almost identical parameters. Further, the experimental data was determined with high precision as the noise-to-signal ratio (defined as the ratio between the standard error and the mean (σ/μ)) was less than 2%. The high precession in the experimental data reduce the likelihood of parameter dependency, as the clear curvature is not lost in the noise. The data obtained in the very early phase of the reaction <10 seconds may have a low accuracy despite a high precision as the time resolution of the biosensor could cause a systematic error that might give a biased estimate of the fast rate-constants (such as k_{cat}). To test this effect on the estimated parameters we simulated a curve using the obtained parameters for *Tre*Cel7A and added a 2.5% random noise (normal distributed) to the data points.

The simulated data was treated as experimental data and fitted to the processive model using non-linear regression analysis. The fit can be seen in Figure S7 and the estimated parameters from the computed experiment can be found in Table S2. As seen from Figure S7 and Table S2 the time resolution of the biosensor may influence the derived parameters. However, such systematic error was similar for all the investigated enzymes. Since the main purpose of the article is to discuss comparative aspects of the investigated enzymes, we argue that the estimated parameters are suitable for this task. To determine the kinetic parameters with higher accuracy, a more a comprehensive pre-steady state kinetic characterization should be done, but this is beyond the scope of this article.

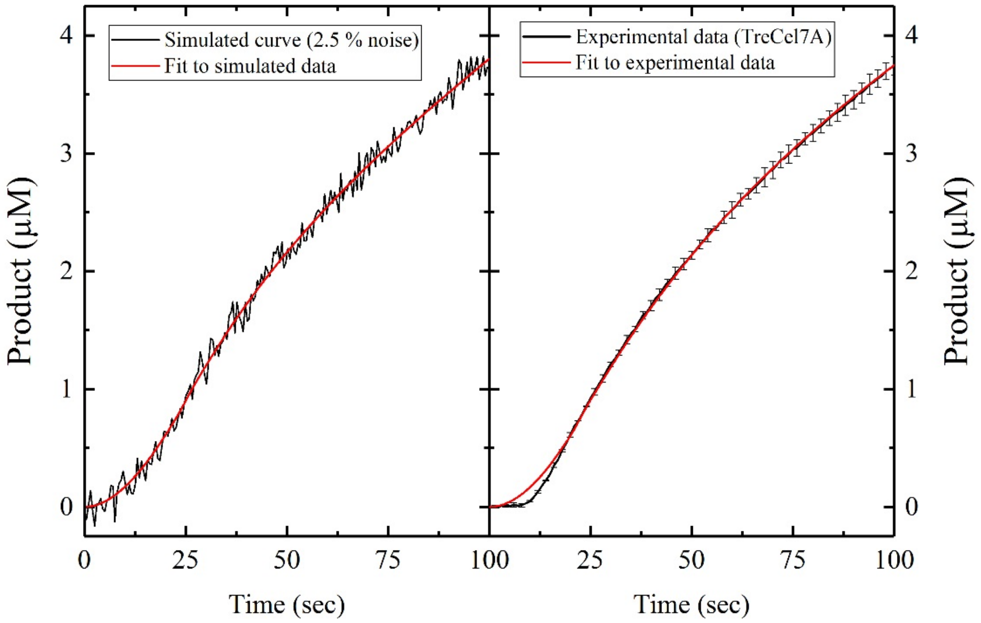

Figure S7. Kinetic parameter fit to simulated data with 2.5 % random noise added (left panel), and to experimental data recorded for *Tre*Cel7A during initial hydrolysis of BMCC (right panel).

Table S2. Comparison of kinetic parameters from the fit to the simulated data with 2.5% noise, and experimental data for *Tre*Cel7A, shown in Figure S7.

**Parameters**	**Fit to experimental data**	**Fit to simulated data (2.5% noise)**	**Ratio**
k_{on} $(g^{-1}Ls^{-1})$	0.0055	0.0075	0.7
k_{cat} (s^{-1})	4.0	3.1	1.3
k_{off} (s^{-1})	0.0066	0.0109	0.6
n	89	77	1.2

Molecular evolution of Cel7A

Figure S8 shows a multiple sequence alignment of the GH7 CBHs used for RCA analysis. Two groups of related fungi within the order Hypocreales were selected for comparison, *Trichoderma* spp. (11 sequences) versus *Fusarium* spp. and *Clonostachys rosea* (6 sequences).

A phylogenetic analysis of GH7 sequences from *Trichoderma* and *Fusarium* spp. confirmed the orthologous status of the Cel7A sequences selected for the RCA analysis (Figure S9).

Table S3 lists the S scores from RCA analysis for residues of interest in *Tat*Cel7A, *Tha*Cel7A, and *Tre*Cel7A.

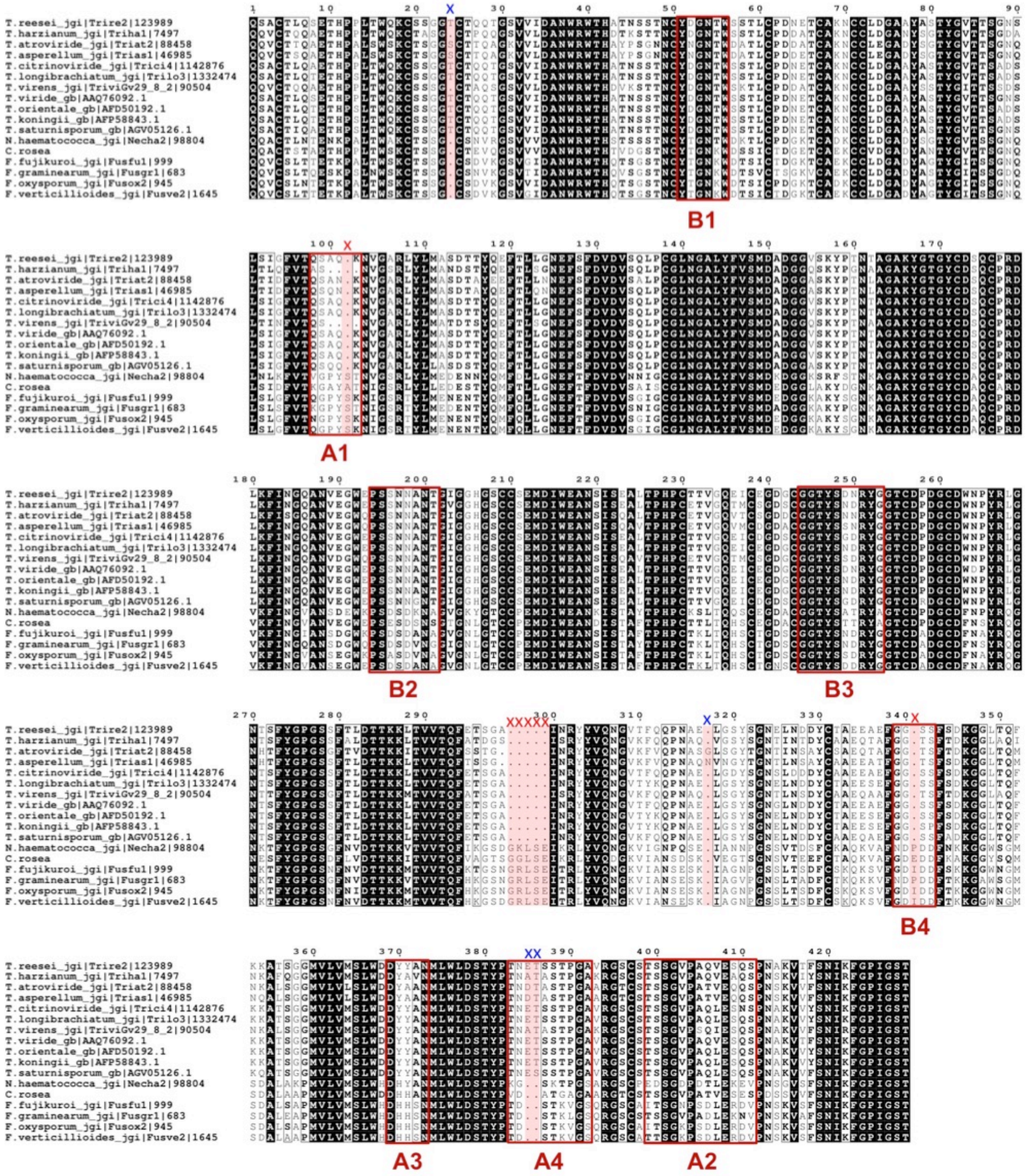

Figure S8. Sequence alignment of GH7 CBH CDs from *Trichoderma* spp. and *Fusarium* spp. 17 amino acid sequences for catalytic domains without signal peptide were aligned with ClustalW using MEGA7. Strictly identical residues are marked in white letters on a black background. Regions of conserved, highly similar residues are framed in thin-lined boxes with bold letters. Red frames indicate loop regions of interest, with loop nomenclature underneath. The light red boxes indicate residue deletion in the alignment used further in RCA. Blue and red “x” labels indicate residue deletion in *Trichoderma* and *Fusarium* spp correspondingly. The figure was prepared using the ESPript web server with default parameters (http://espript.ibcp.fr;(12)).

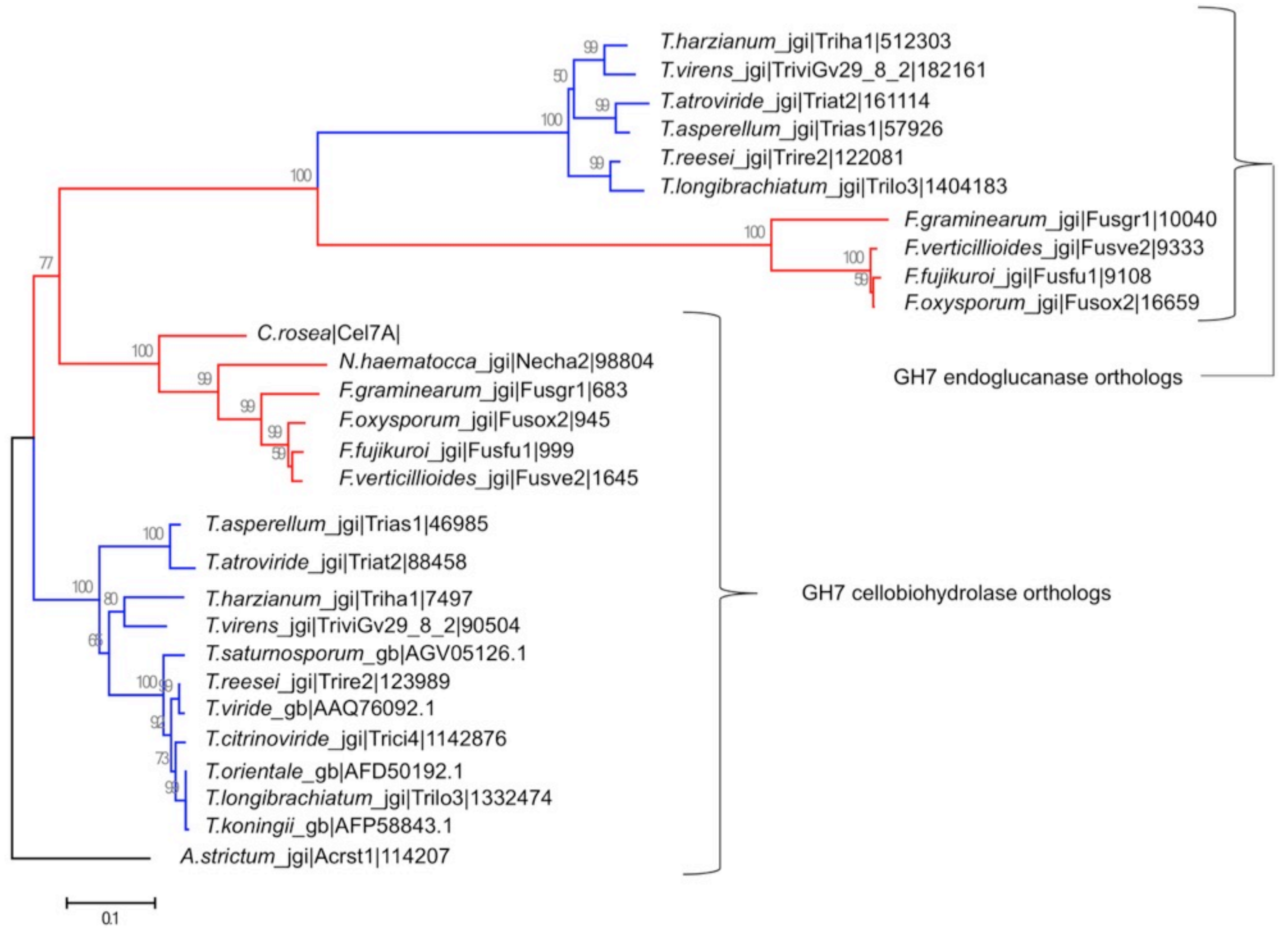

Figure S9. Phylogenetic tree of GH7 catalytic domain protein sequences from *Trichoderma* spp and *Fusarium* spp. The evolutionary history was inferred using the minimum evolution (ME) method (6). The optimal tree with the sum of branch length = 2.92 is shown. The percentage of replicate trees in which the associated taxa clustered together in the bootstrap test (2000 replicates) are shown above the branches (7). The tree is drawn to scale, with branch lengths in the same units as those of the evolutionary distances used to infer the phylogenetic tree. The evolutionary distances were computed using the Dayhoff matrix based method (8) and are in the units of the number of amino acid substitutions per site. The ME tree was searched using the Close-Neighbor-Interchange (CNI) algorithm (9) at a search level of 1. The Neighbor-joining algorithm (10) was used to generate the initial tree. The analysis involved 28 amino acid sequences. All positions containing gaps and missing data were eliminated. There were a total of 349 positions in the final dataset. Evolutionary analyses were conducted in MEGA7 (11).

Table S3. S scores for residues of interest for *Tat*Cel7A, *Tha*Cel7A and *Tre*Cel7A

Section	S score	***Tat*Cel7A**	***Tha*Cel7A**	***Tre*Cel7A**
I	2.2721	Gly88	Gly88	Gly87
	1.7632	Asn89	Asp88	Asn89
	1.6409	Gln90	Ala89	Ser90
II	1.1766	Val236	Thr232	Glu236
	1.5883	Thr237	Met233	Ile237
	3.2713	Ser239	Ser235	Glu239
	3.0538	Asp242	Ser238	Gly242
III	2.1341	Ser316	Gln313	Glu317
	-	Gly317	-	-
	2.4929	Lue318	Val314	Leu318
	2.9897	Ser319	Gly315	Gly319
	2.0726	Gly320	Ser316	Ser320
IV	1.4789	Met352	Ile348	Phe342
	2.6619	Lys354	Lys350	Lys354
	1.4963	Leu356	Phe352	Thr356

Molecular dynamics simulation results

The root mean square deviation (RMSD) of the protein backbone atoms from the proteins' initial positions in the data collection simulation (i.e., immediately following the density equilibration period) as a function of time was determined for each 100-ns simulation (Figure S10). The proteins are relatively stable over the course of each simulation and do not undergo any major conformational change from their initial positions. The RMSD of each plateaus quickly, after ~20 ns, indicating the simulations converged upon a local equilibrium.

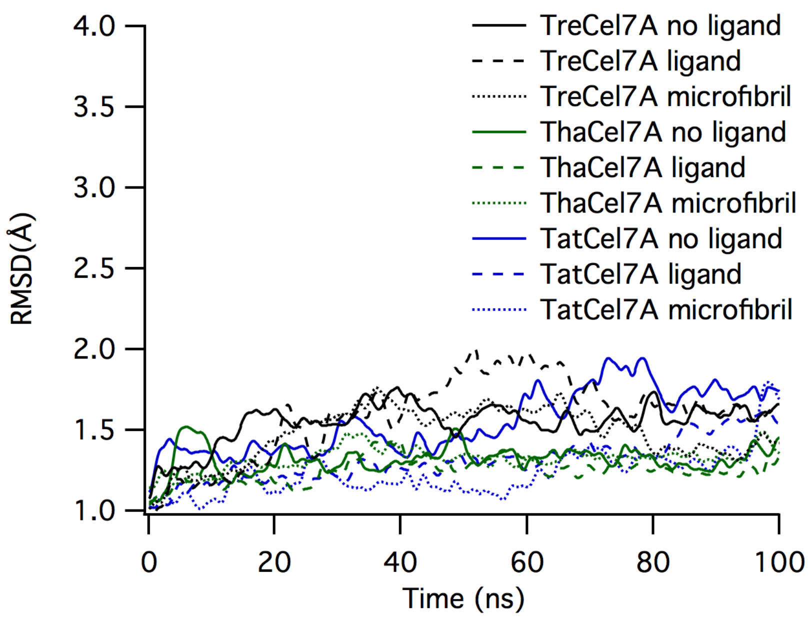

Figure S10. RMSD as a function of time for each 100-ns, ligand-bound MD simulation of *Tre*Cel7A, *Tha*Cel7A, and *Tat*CelA, labeled as shown.

References

1. Zhang YH & Lynd LR (2004) Toward an aggregated understanding of enzymatic hydrolysis of cellulose: noncomplexed cellulase systems. Biotechnology and bioengineering 88(7):797-824.
2. Mansfield SD, Mooney C, & Saddler JN (1999) Substrate and Enzyme Characteristics that Limit Cellulose Hydrolysis. Biotechnol Prog 15(5):804-816.
3. Kipper K, Väljamäe P, & Johansson G (2005) Processive action of cellobiohydrolase Cel7A from Trichoderma reesei is revealed as 'burst' kinetics on fluorescent polymeric model substrates. Biochem. J. 385(2):527-535.
4. Murphy L, et al. (2012) Origin of initial burst in activity for Trichoderma reesei endo-glucanases hydrolyzing insoluble cellulose. The Journal of biological chemistry 287(2):1252-1260.
5. Praestgaard E, et al. (2011) A kinetic model for the burst phase of processive cellulases. The FEBS journal 278(9):1547-1560.
6. Rzhetsky A & Nei M (1992) Statistical properties of the ordinary least-squares, generalized least-squares, and minimum-evolution methods of phylogenetic inference. Journal of molecular evolution 35(4):367-375.
7. Felsenstein J (1985) Confidence limits on phylogenies: an approach using the bootstrap. Evolution:783-791.
8. Schwartz R & Dayhoff M (1978) Matrices for detecting distant relationships. Atlas of protein sequence and structure 5(suppl 3):353-358.
9. Nei M & Kumar S (2000) Molecular evolution and phylogenetics (Oxford university press).
10. Saitou N & Nei M (1987) The neighbor-joining method: a new method for reconstructing phylogenetic trees. Molecular biology and evolution 4(4):406-425.
11. Kumar S, Stecher G, & Tamura K (2016) MEGA7: Molecular Evolutionary Genetics Analysis version 7.0 for bigger datasets. Molecular Biology and Evolution accepted.
12. Robert X & Gouet P (2014) Deciphering key features in protein structures with the new ENDscript server. Nucleic acids research 42(Web Server issue):W320-324.
